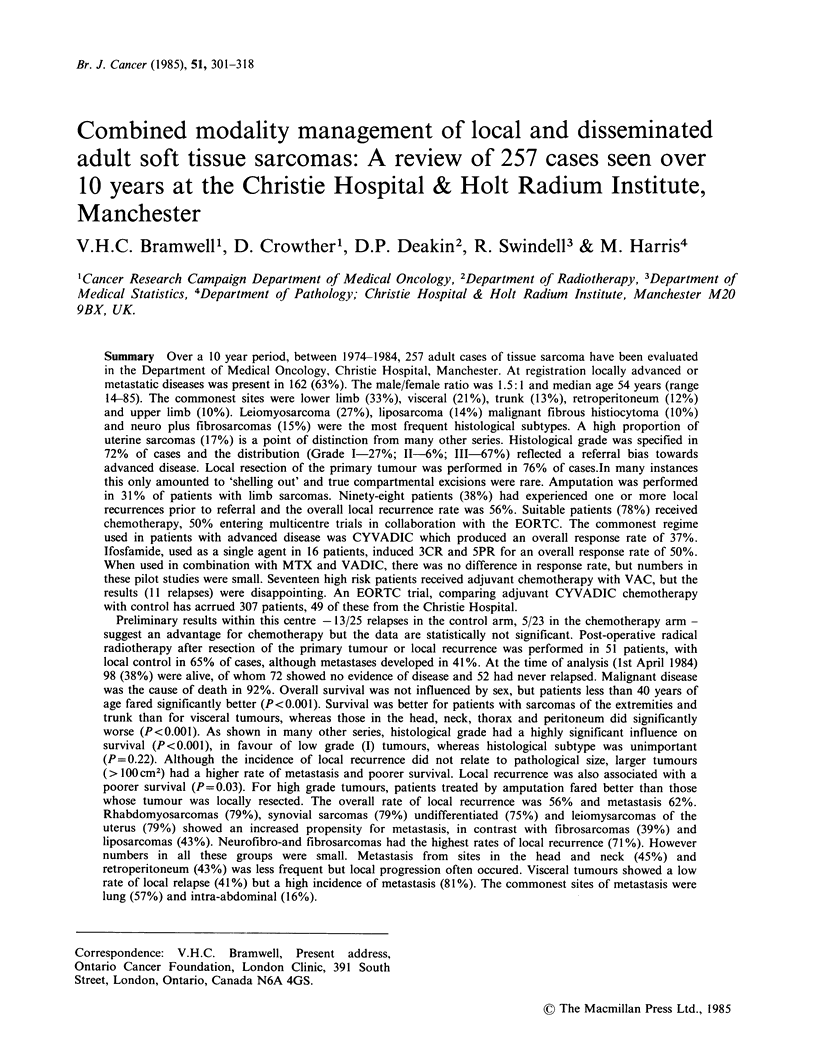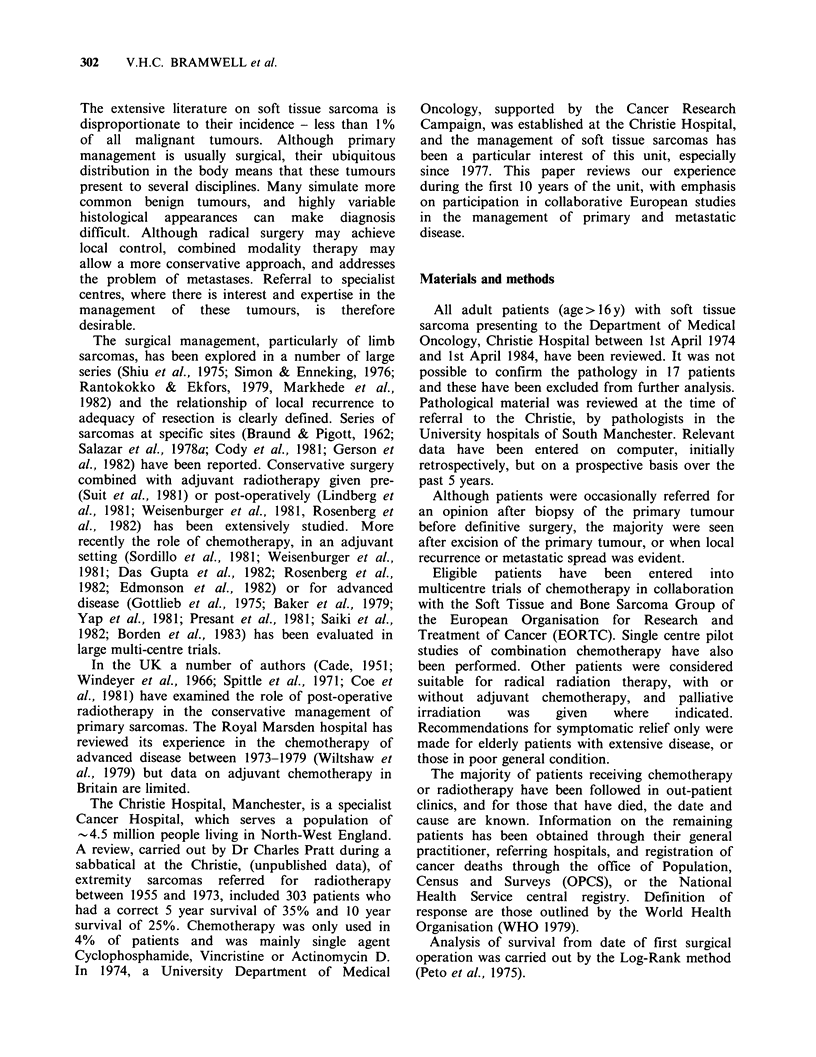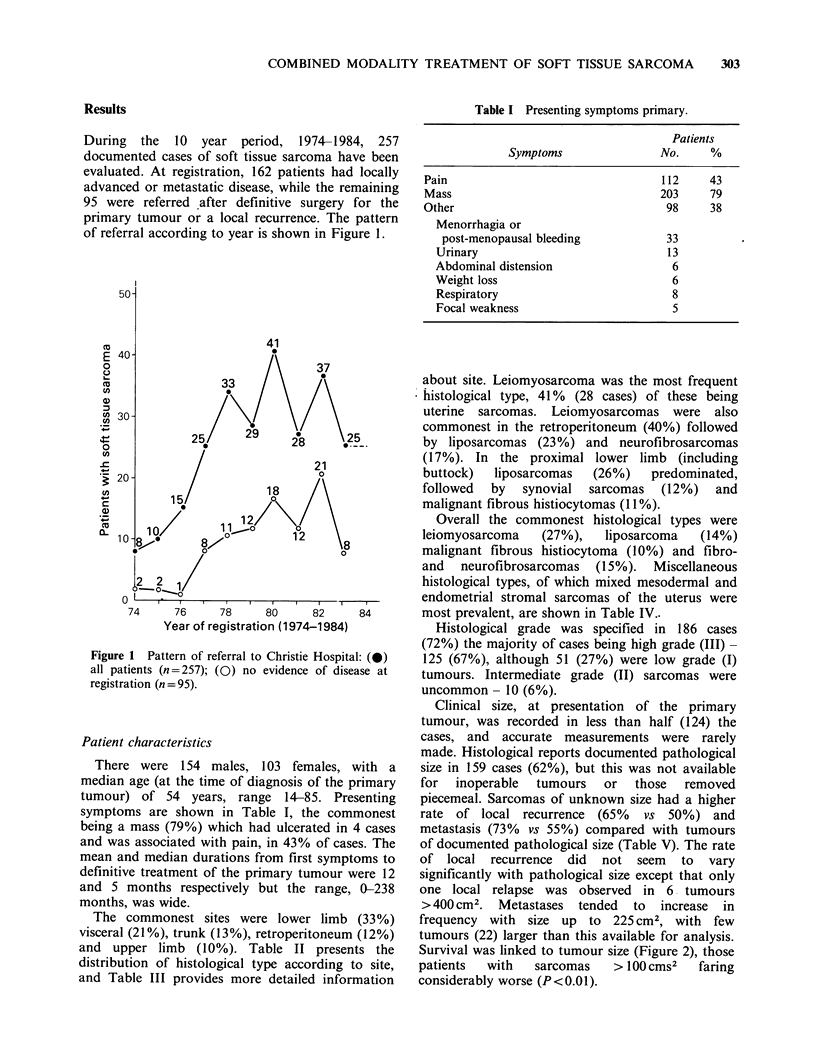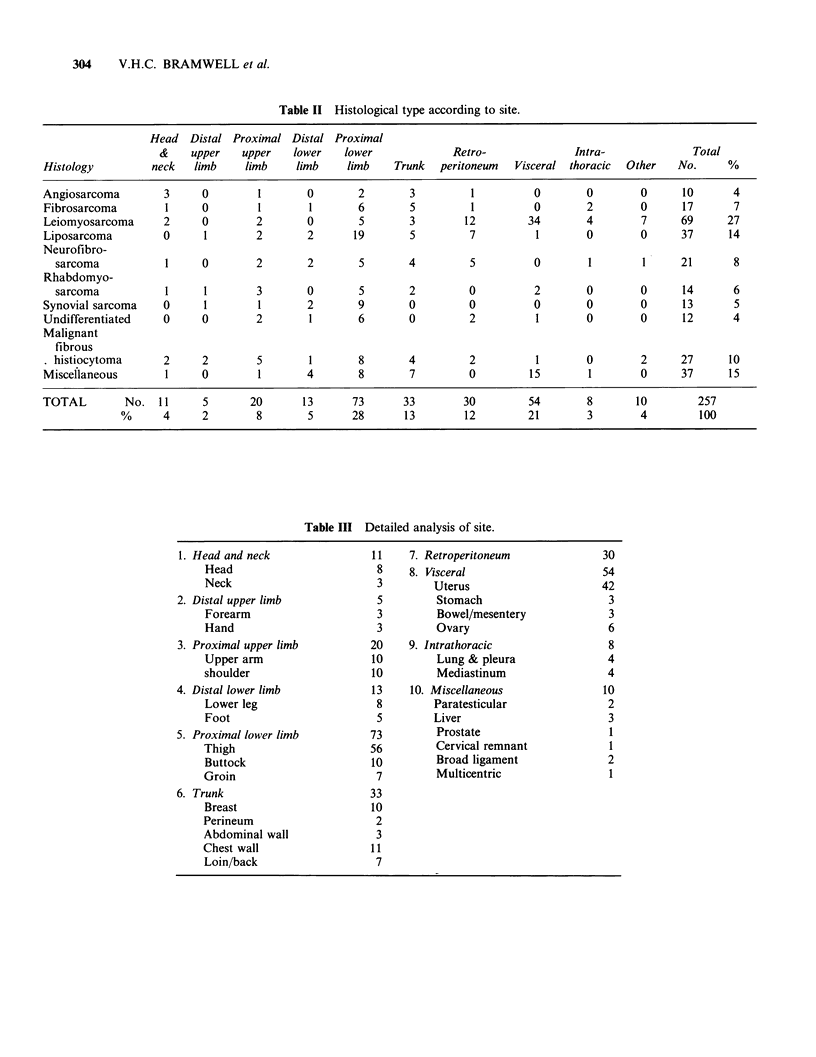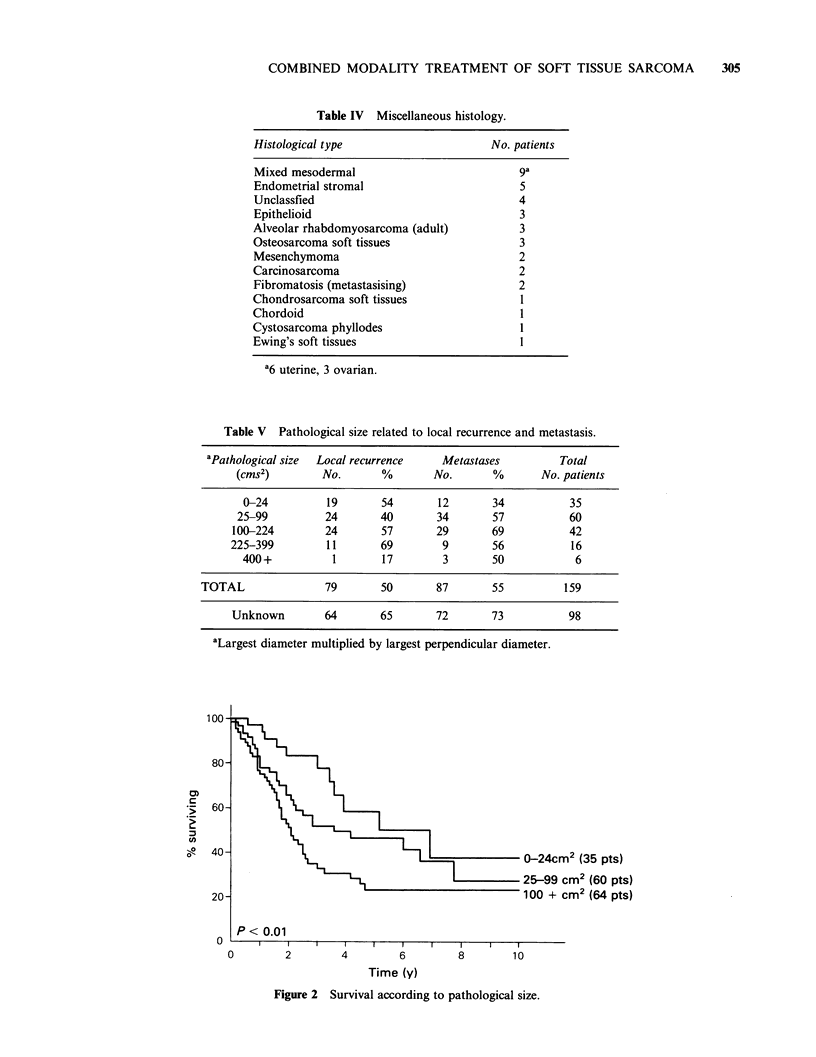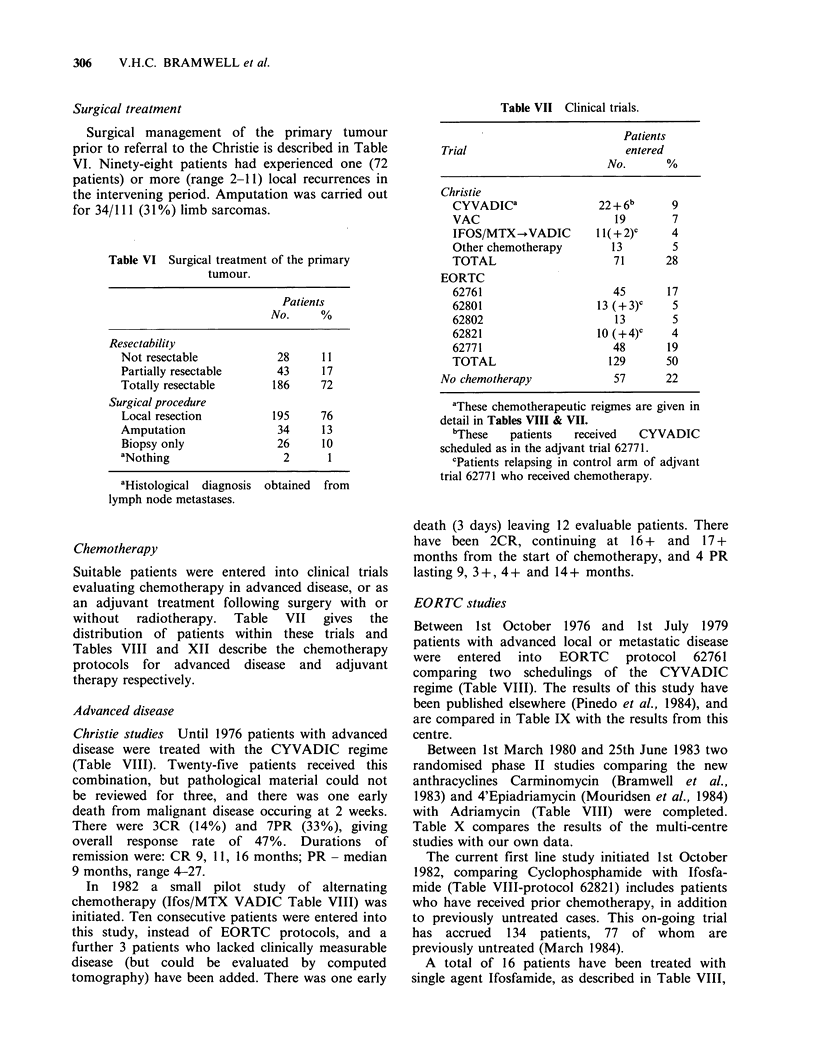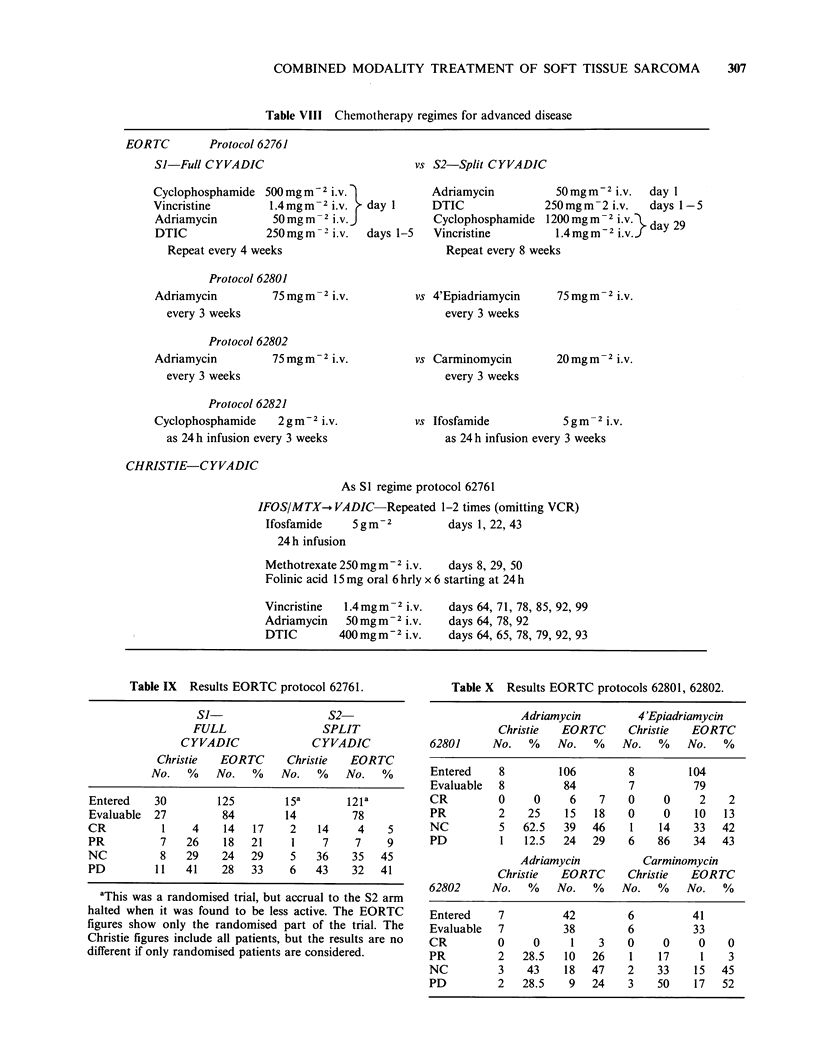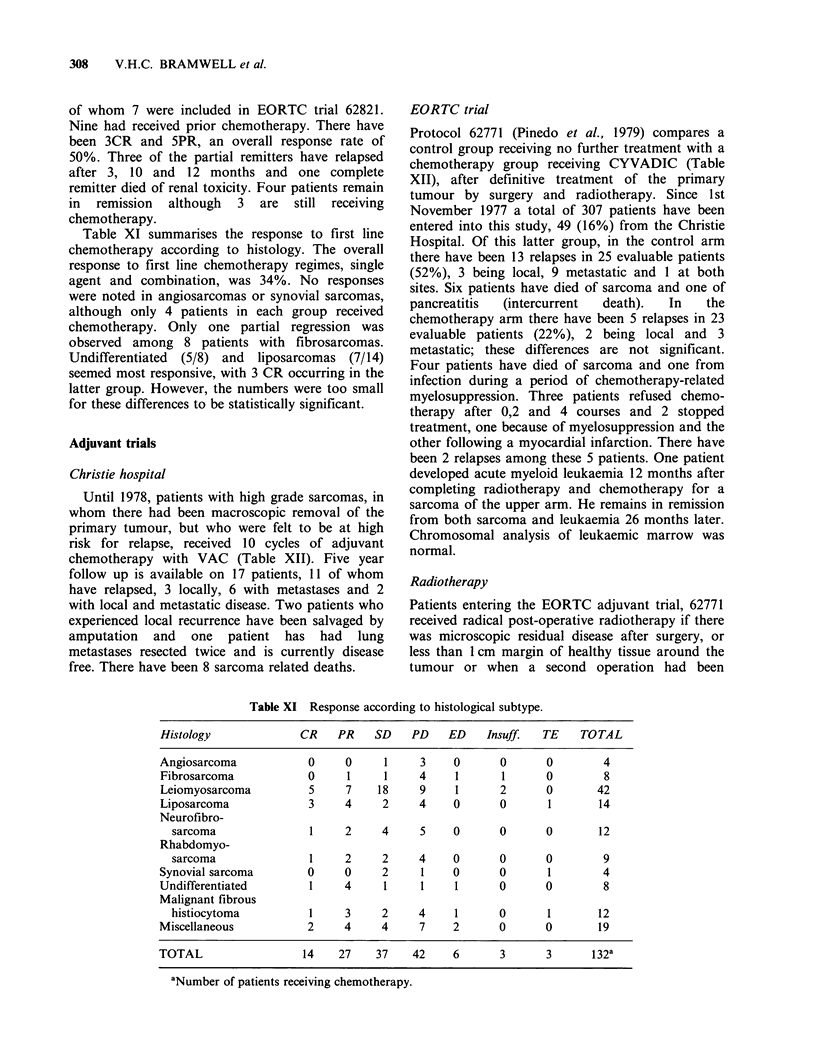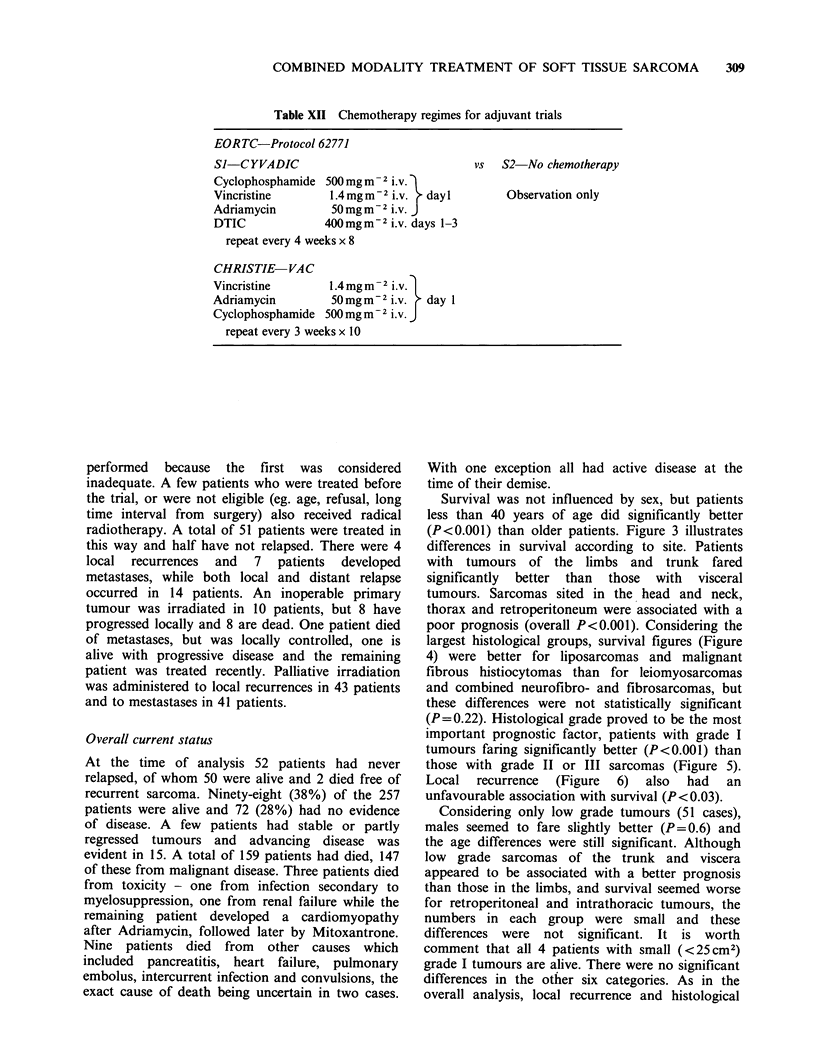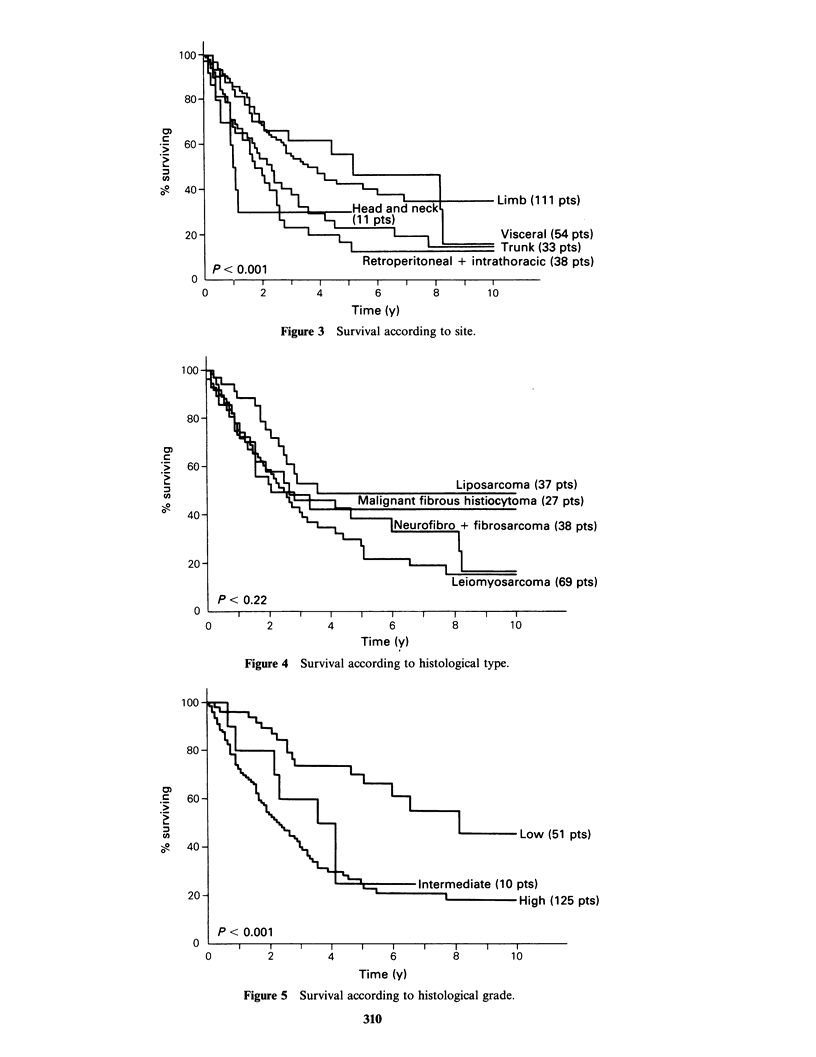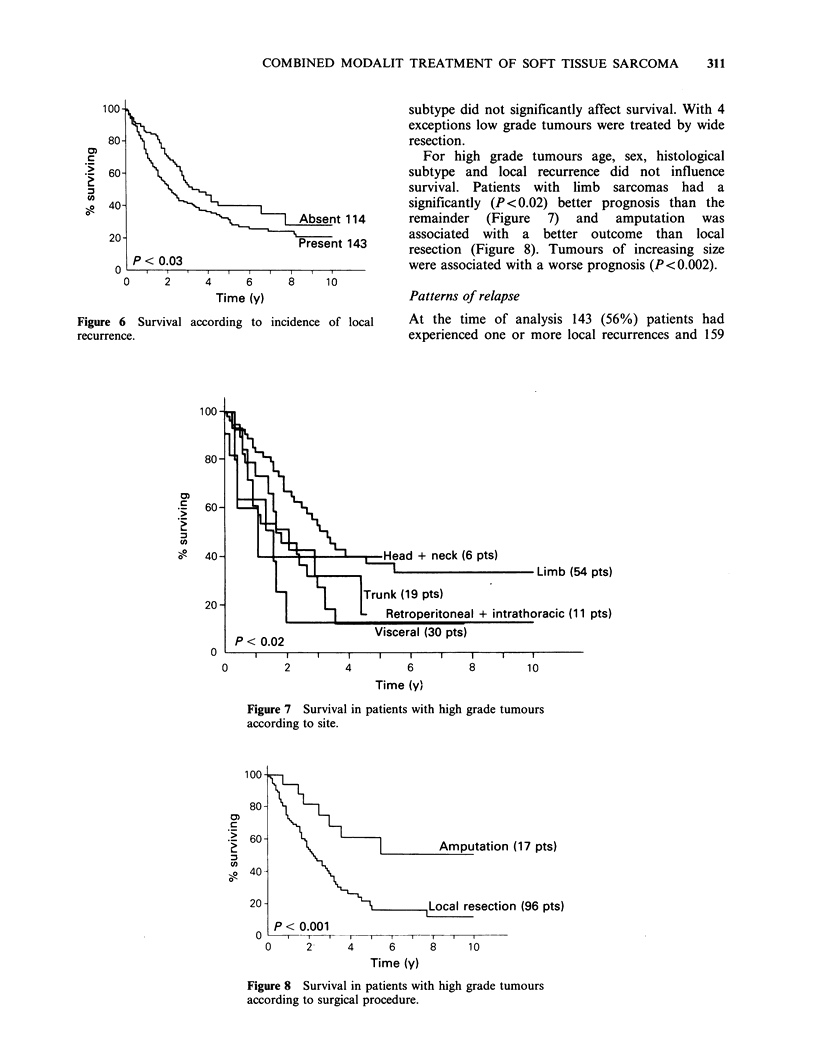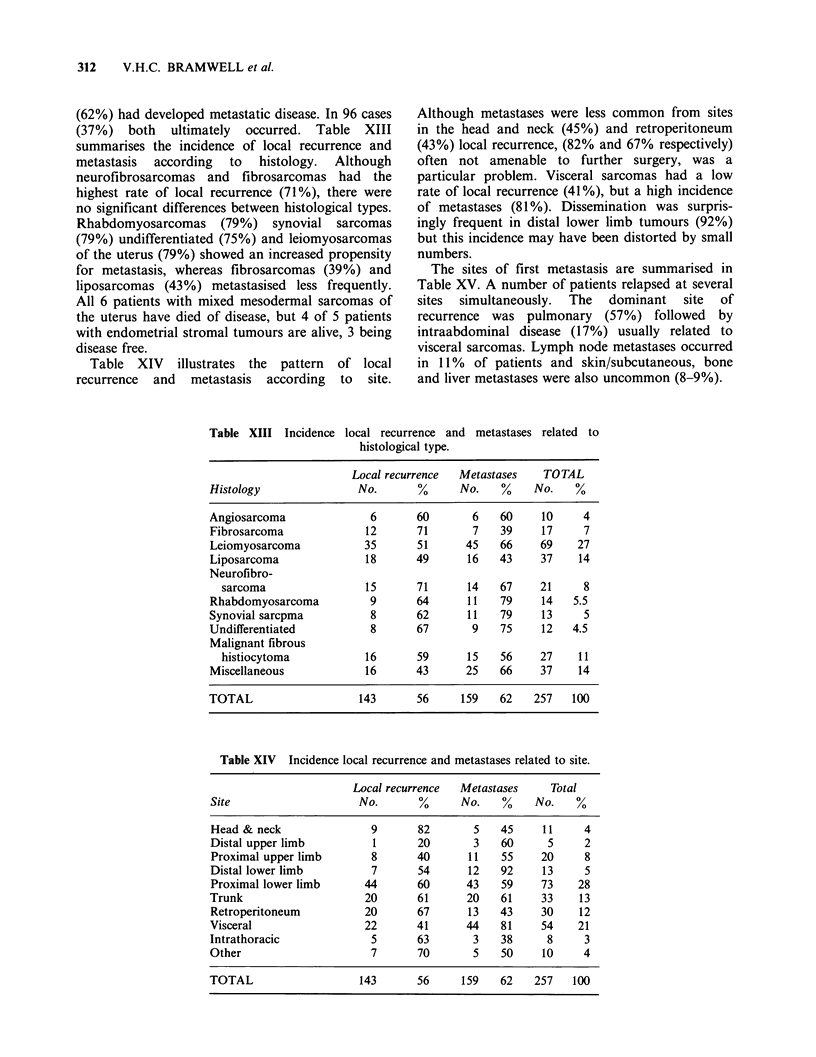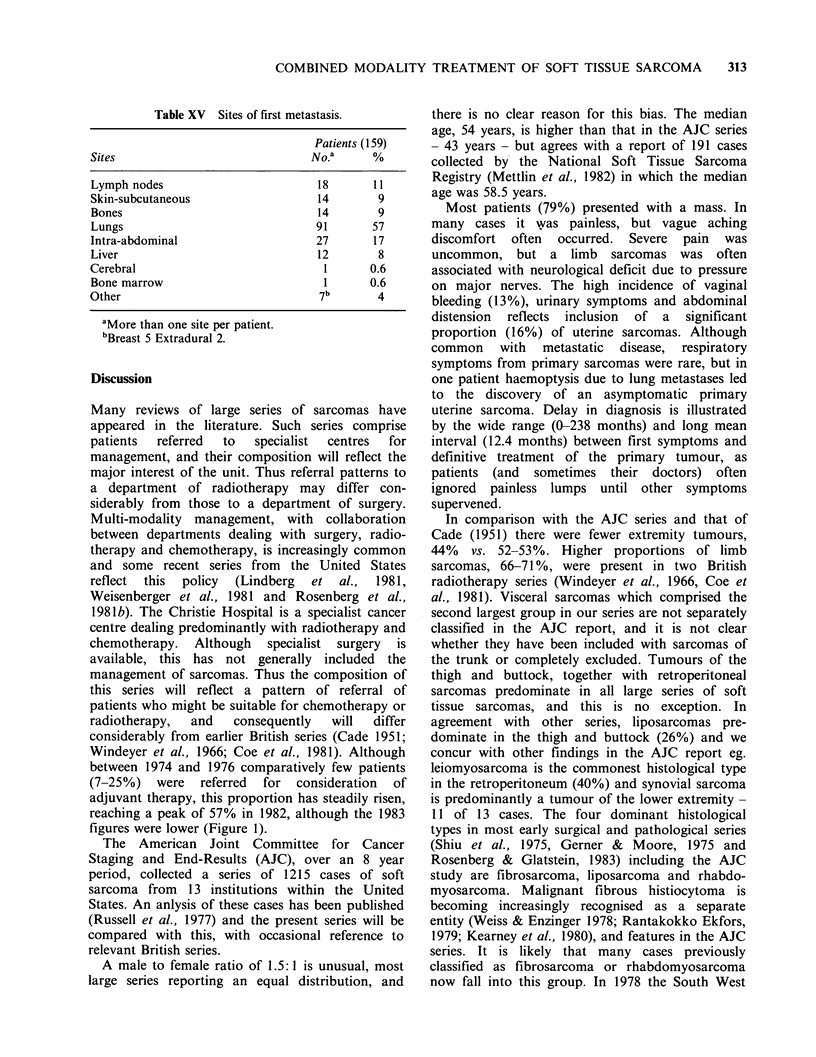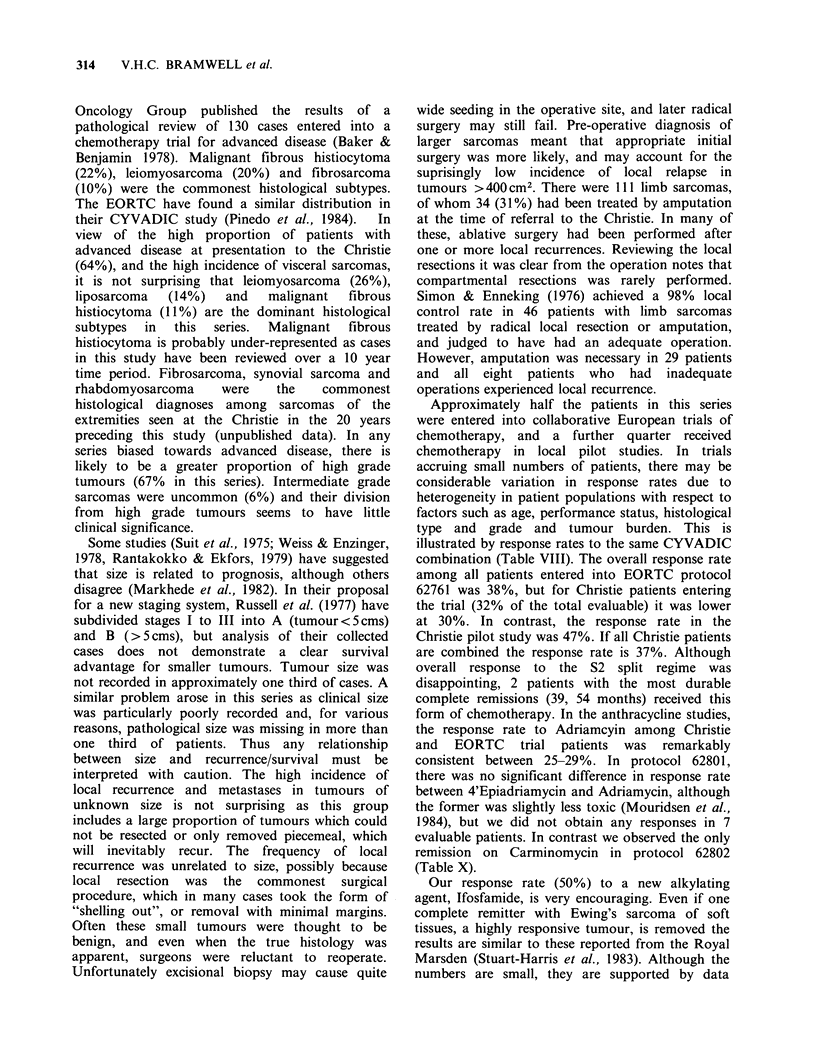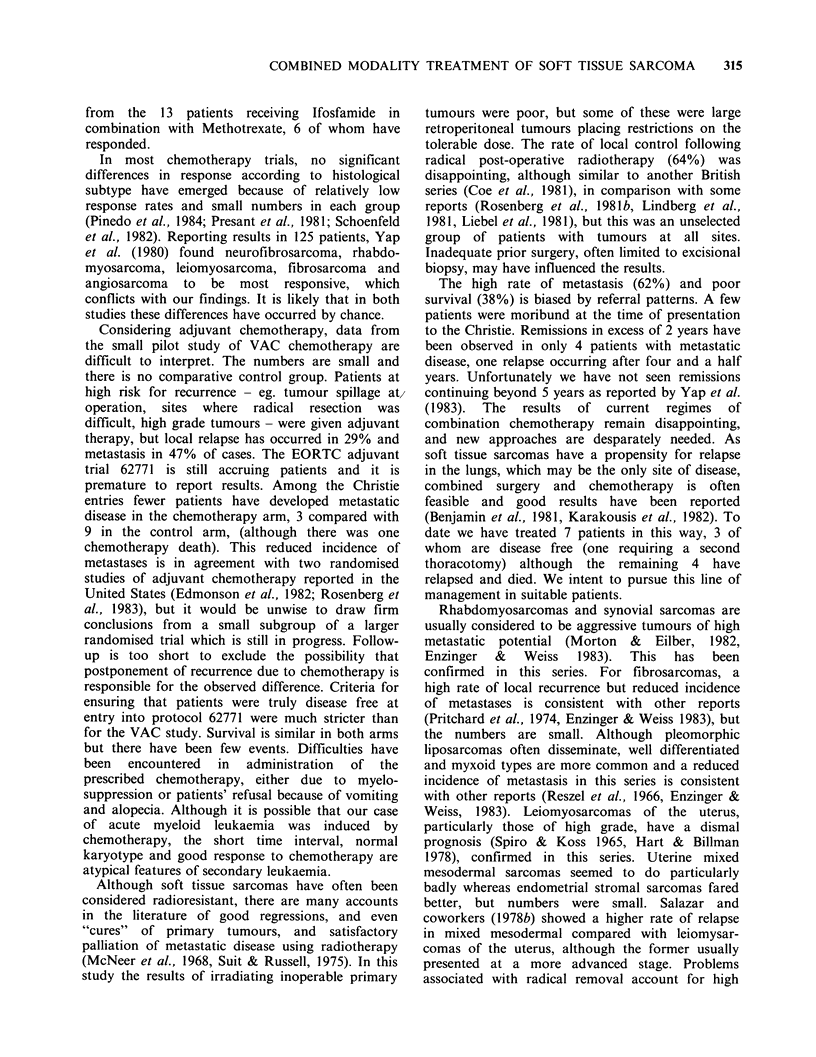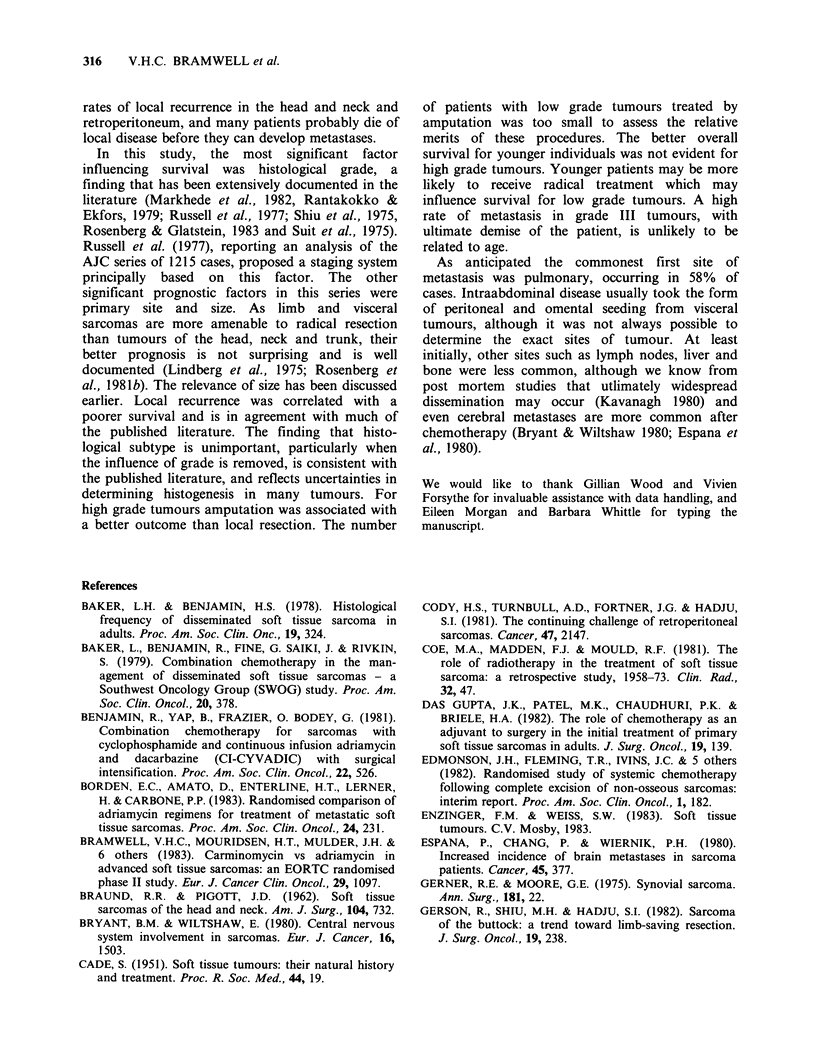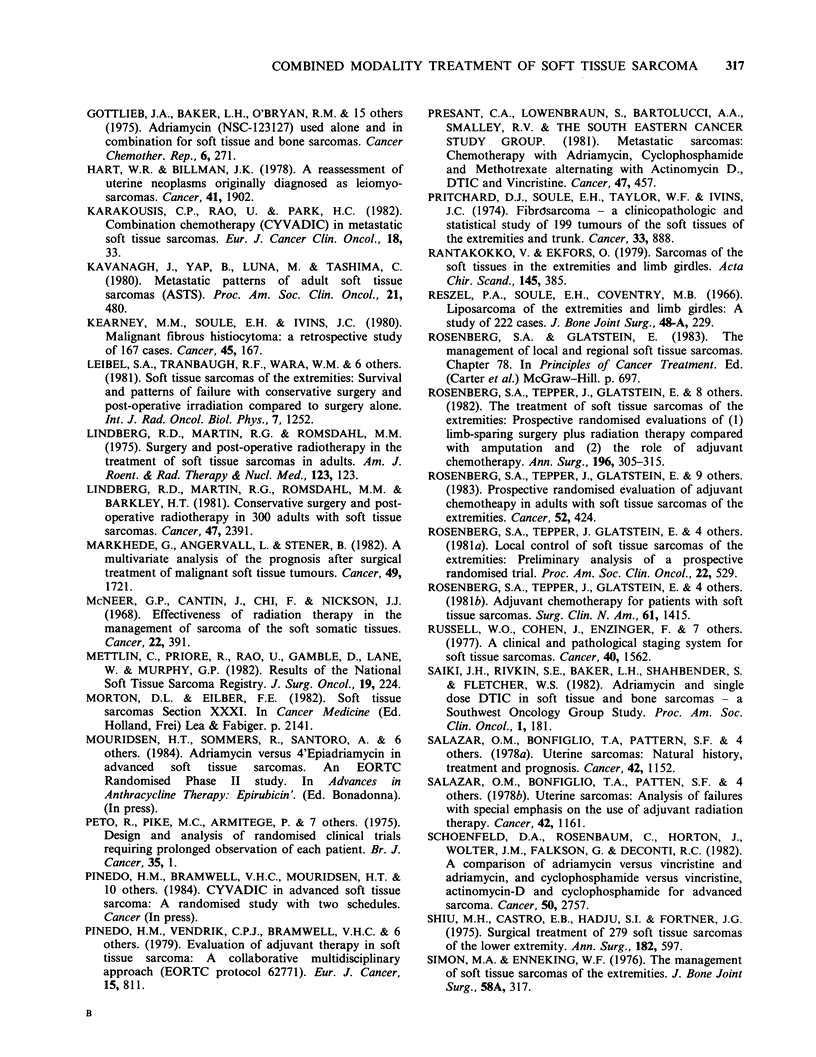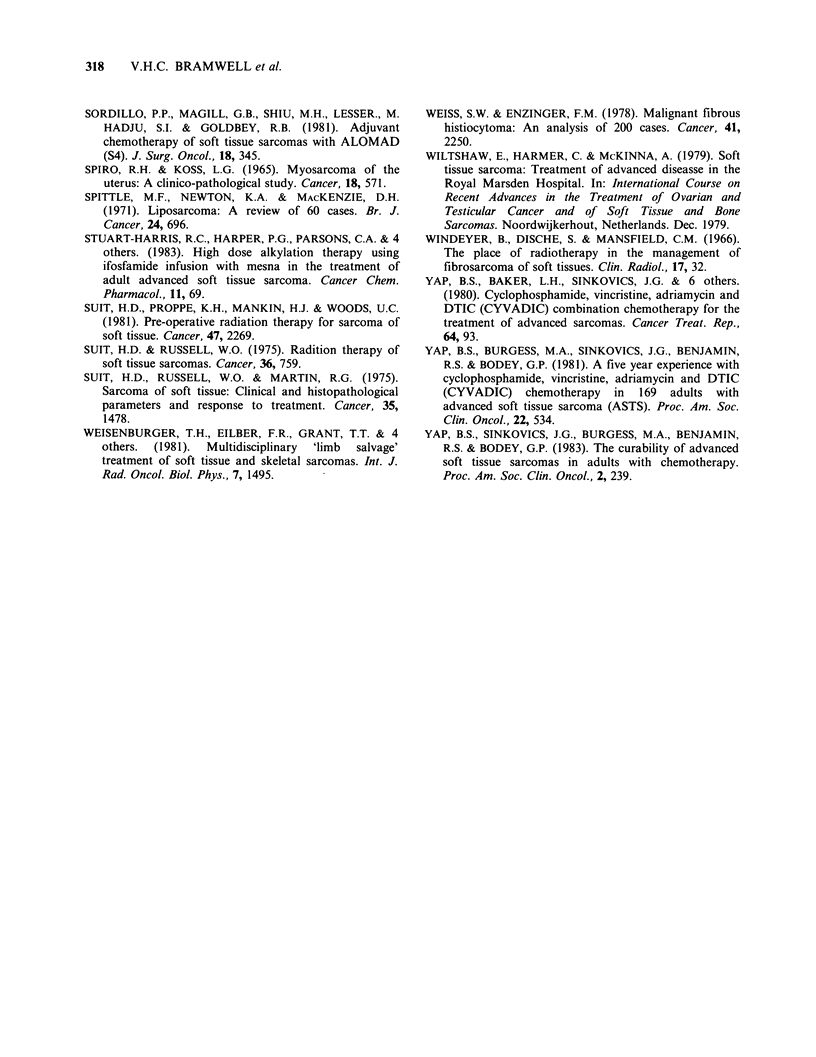# Combined modality management of local and disseminated adult soft tissue sarcomas: a review of 257 cases seen over 10 years at the Christie Hospital & Holt Radium Institute, Manchester.

**DOI:** 10.1038/bjc.1985.43

**Published:** 1985-03

**Authors:** V. H. Bramwell, D. Crowther, D. P. Deakin, R. Swindell, M. Harris

## Abstract

Over a 10 year period, between 1974-1984, 257 adult cases of tissue sarcoma have been evaluated in the Department of Medical Oncology, Christie Hospital, Manchester. At registration locally advanced or metastatic diseases was present in 162 (63%). The male/female ratio was 1.5:1 and median age 54 years (range 14-85). The commonest sites were lower limb (33%), visceral (21%), trunk (13%), retroperitoneum (12%) and upper limb (10%). Leiomyosarcoma (27%), liposarcoma (14%) malignant fibrous histiocytoma (10%) and neuro plus fibrosarcomas (15%) were the most frequent histological subtypes. A high proportion of uterine sarcomas (17%) is a point of distinction from many other series. Histological grade was specified in 72% of cases and the distribution (Grade I--27%; II--6%; III--67%) reflected a referral bias towards advanced disease. Local resection of the primary tumour was performed in 76% of cases. In many instances this only amounted to 'shelling out' and true compartmental excisions were rare. Amputation was performed in 31% of patients with limb sarcomas. Ninety-eight patients (38%) had experienced one or more local recurrences prior to referral and the overall local recurrence rate was 56%. Suitable patients (78%) received chemotherapy, 50% entering multicentre trials in collaboration with the EORTC. The commonest regime used in patients with advanced disease was CYVADIC which produced an overall response rate of 37%. Ifosfamide, used as a single agent in 16 patients, induced 3CR and 5PR for an overall response rate of 50%. When used in combination with MTX and VADIC, there was no difference in response rate, but numbers in these pilot studies were small. Seventeen high risk patients received adjuvant chemotherapy with VAC, but the results (11 relapses) were disappointing. An EORTC trial, comparing adjuvant CYVADIC chemotherapy with control has accrued 307 patients, 49 of these from the Christie Hospital. Preliminary results within this centre - 13/25 relapses in the control arm, 5/23 in the chemotherapy arm-suggest an advantage for chemotherapy but the data are statistically not significant. Post-operative radical radiotherapy after resection of the primary tumour or local recurrence was performed in 51 patients, with local control in 65% of cases, although metastases developed in 41%. At the time of analysis (1st April 1984) 98 (38%) were alive, of whom 72 showed no evidence of disease and 52 had never relapsed. Malignant disease was the cause of death in 92%. Overall survival was not influenced by sex, but patients less than 40 years of age fared significantly better (P less than 0.001).(ABSTRACT TRUNCATED AT 400 WORDS)


Br. J. Cancer (1985), 51, 301–318

### Combined modality management of local and disseminated adult soft tissue sarcomas: A review of 257 cases seen over 10 years at the Christie Hospital & Holt Radium Institute, Manchester

V.H.C. Bramwell[1], D. Crowther[1], D.P. Deakin[2], R. Swindell[3] & M. Harris[4]

[1]*Cancer Research Campaign Department of Medical Oncology,* [2]*Department of Radiotherapy,* [3]*Department of Medical Statistics,* [4]*Department of Pathology; Christie Hospital & Holt Radium Institute, Manchester M20 9BX, UK.*

**Summary** Over a 10 year period, between 1974–1984, 257 adult cases of tissue sarcoma have been evaluated in the Department of Medical Oncology, Christie Hospital, Manchester. At registration locally advanced or metastatic diseases was present in 162 (63%). The male/female ratio was 1.5:1 and median age 54 years (range 14–85). The commonest sites were lower limb (33%), visceral (21%), trunk (13%), retroperitoneum (12%) and upper limb (10%). Leiomyosarcoma (27%), liposarcoma (14%) malignant fibrous histiocytoma (10%) and neuro plus fibrosarcomas (15%) were the most frequent histological subtypes. A high proportion of uterine sarcomas (17%) is a point of distinction from many other series. Histological grade was specified in 72% of cases and the distribution (Grade I—27%; II—6%; III—67%) reflected a referral bias towards advanced disease. Local resection of the primary tumour was performed in 76% of cases.In many instances this only amounted to 'shelling out' and true compartmental excisions were rare. Amputation was performed in 31% of patients with limb sarcomas. Ninety-eight patients (38%) had experienced one or more local recurrences prior to referral and the overall local recurrence rate was 56%. Suitable patients (78%) received chemotherapy, 50% entering multicentre trials in collaboration with the EORTC. The commonest regime used in patients with advanced disease was CYVADIC which produced an overall response rate of 37%. Ifosfamide, used as a single agent in 16 patients, induced 3CR and 5PR for an overall response rate of 50%. When used in combination with MTX and VADIC, there was no difference in response rate, but numbers in these pilot studies were small. Seventeen high risk patients received adjuvant chemotherapy with VAC, but the results (11 relapses) were disappointing. An EORTC trial, comparing adjuvant CYVADIC chemotherapy with control has acrrued 307 patients, 49 of these from the Christie Hospital.

Preliminary results within this centre – 13/25 relapses in the control arm, 5/23 in the chemotherapy arm – suggest an advantage for chemotherapy but the data are statistically not significant. Post-operative radical radiotherapy after resection of the primary tumour or local recurrence was performed in 51 patients, with local control in 65% of cases, although metastases developed in 41%. At the time of analysis (1st April 1984) 98 (38%) were alive, of whom 72 showed no evidence of disease and 52 had never relapsed. Malignant disease was the cause of death in 92%. Overall survival was not influenced by sex, but patients less than 40 years of age fared significantly better ($P<0.001$). Survival was better for patients with sarcomas of the extremities and trunk than for visceral tumours, whereas those in the head, neck, thorax and peritoneum did significantly worse ($P<0.001$). As shown in many other series, histological grade had a highly significant influence on survival ($P<0.001$), in favour of low grade (I) tumours, whereas histological subtype was unimportant ($P=0.22$). Although the incidence of local recurrence did not relate to pathological size, larger tumours ($>100\,cm^2$) had a higher rate of metastasis and poorer survival. Local recurrence was also associated with a poorer survival ($P=0.03$). For high grade tumours, patients treated by amputation fared better than those whose tumour was locally resected. The overall rate of local recurrence was 56% and metastasis 62%. Rhabdomyosarcomas (79%), synovial sarcomas (79%) undifferentiated (75%) and leiomysarcomas of the uterus (79%) showed an increased propensity for metastasis, in contrast with fibrosarcomas (39%) and liposarcomas (43%). Neurofibro-and fibrosarcomas had the highest rates of local recurrence (71%). However numbers in all these groups were small. Metastasis from sites in the head and neck (45%) and retroperitoneum (43%) was less frequent but local progression often occured. Visceral tumours showed a low rate of local relapse (41%) but a high incidence of metastasis (81%). The commonest sites of metastasis were lung (57%) and intra-abdominal (16%).

Correspondence: V.H.C. Bramwell, Present address, Ontario Cancer Foundation, London Clinic, 391 South Street, London, Ontario, Canada N6A 4GS.

© The Macmillan Press Ltd., 1985

The extensive literature on soft tissue sarcoma is disproportionate to their incidence – less than 1% of all malignant tumours. Although primary management is usually surgical, their ubiquitous distribution in the body means that these tumours present to several disciplines. Many simulate more common benign tumours, and highly variable histological appearances can make diagnosis difficult. Although radical surgery may achieve local control, combined modality therapy may allow a more conservative approach, and addresses the problem of metastases. Referral to specialist centres, where there is interest and expertise in the management of these tumours, is therefore desirable.

The surgical management, particularly of limb sarcomas, has been explored in a number of large series (Shiu *et al.*, 1975; Simon & Enneking, 1976; Rantokokko & Ekfors, 1979, Markhede *et al.*, 1982) and the relationship of local recurrence to adequacy of resection is clearly defined. Series of sarcomas at specific sites (Braund & Pigott, 1962; Salazar *et al.*, 1978*a*; Cody *et al.*, 1981; Gerson *et al.*, 1982) have been reported. Conservative surgery combined with adjuvant radiotherapy given pre- (Suit *et al.*, 1981) or post-operatively (Lindberg *et al.*, 1981; Weisenburger *et al.*, 1981, Rosenberg *et al.*, 1982) has been extensively studied. More recently the role of chemotherapy, in an adjuvant setting (Sordillo *et al.*, 1981; Weisenburger *et al.*, 1981; Das Gupta *et al.*, 1982; Rosenberg *et al.*, 1982; Edmonson *et al.*, 1982) or for advanced disease (Gottlieb *et al.*, 1975; Baker *et al.*, 1979; Yap *et al.*, 1981; Presant *et al.*, 1981; Saiki *et al.*, 1982; Borden *et al.*, 1983) has been evaluated in large multi-centre trials.

In the UK a number of authors (Cade, 1951; Windeyer *et al.*, 1966; Spittle *et al.*, 1971; Coe *et al.*, 1981) have examined the role of post-operative radiotherapy in the conservative management of primary sarcomas. The Royal Marsden hospital has reviewed its experience in the chemotherapy of advanced disease between 1973–1979 (Wiltshaw *et al.*, 1979) but data on adjuvant chemotherapy in Britain are limited.

The Christie Hospital, Manchester, is a specialist Cancer Hospital, which serves a population of ~4.5 million people living in North-West England. A review, carried out by Dr Charles Pratt during a sabbatical at the Christie, (unpublished data), of extremity sarcomas referred for radiotherapy between 1955 and 1973, included 303 patients who had a correct 5 year survival of 35% and 10 year survival of 25%. Chemotherapy was only used in 4% of patients and was mainly single agent Cyclophosphamide, Vincristine or Actinomycin D. In 1974, a University Department of Medical Oncology, supported by the Cancer Research Campaign, was established at the Christie Hospital, and the management of soft tissue sarcomas has been a particular interest of this unit, especially since 1977. This paper reviews our experience during the first 10 years of the unit, with emphasis on participation in collaborative European studies in the management of primary and metastatic disease.

### Materials and methods

All adult patients (age > 16 y) with soft tissue sarcoma presenting to the Department of Medical Oncology, Christie Hospital between 1st April 1974 and 1st April 1984, have been reviewed. It was not possible to confirm the pathology in 17 patients and these have been excluded from further analysis. Pathological material was reviewed at the time of referral to the Christie, by pathologists in the University hospitals of South Manchester. Relevant data have been entered on computer, initially retrospectively, but on a prospective basis over the past 5 years.

Although patients were occasionally referred for an opinion after biopsy of the primary tumour before definitive surgery, the majority were seen after excision of the primary tumour, or when local recurrence or metastatic spread was evident.

Eligible patients have been entered into multicentre trials of chemotherapy in collaboration with the Soft Tissue and Bone Sarcoma Group of the European Organisation for Research and Treatment of Cancer (EORTC). Single centre pilot studies of combination chemotherapy have also been performed. Other patients were considered suitable for radical radiation therapy, with or without adjuvant chemotherapy, and palliative irradiation was given where indicated. Recommendations for symptomatic relief only were made for elderly patients with extensive disease, or those in poor general condition.

The majority of patients receiving chemotherapy or radiotherapy have been followed in out-patient clinics, and for those that have died, the date and cause are known. Information on the remaining patients has been obtained through their general practitioner, referring hospitals, and registration of cancer deaths through the office of Population, Census and Surveys (OPCS), or the National Health Service central registry. Definition of response are those outlined by the World Health Organisation (WHO 1979).

Analysis of survival from date of first surgical operation was carried out by the Log-Rank method (Peto *et al.*, 1975).

### Results

During the 10 year period, 1974–1984, 257 documented cases of soft tissue sarcoma have been evaluated. At registration, 162 patients had locally advanced or metastatic disease, while the remaining 95 were referred after definitive surgery for the primary tumour or a local recurrence. The pattern of referral according to year is shown in Figure 1.

**Figure 1** Pattern of referral to Christie Hospital: (●) all patients ($n=257$); (○) no evidence of disease at registration ($n=95$).

### *Patient characteristics*

There were 154 males, 103 females, with a median age (at the time of diagnosis of the primary tumour) of 54 years, range 14–85. Presenting symptoms are shown in Table I, the commonest being a mass (79%) which had ulcerated in 4 cases and was associated with pain, in 43% of cases. The mean and median durations from first symptoms to definitive treatment of the primary tumour were 12 and 5 months respectively but the range, 0–238 months, was wide.

The commonest sites were lower limb (33%) visceral (21%), trunk (13%), retroperitoneum (12%) and upper limb (10%). Table II presents the distribution of histological type according to site, and Table III provides more detailed information about site. Leiomyosarcoma was the most frequent histological type, 41% (28 cases) of these being uterine sarcomas. Leiomyosarcomas were also commonest in the retroperitoneum (40%) followed by liposarcomas (23%) and neurofibrosarcomas (17%). In the proximal lower limb (including buttock) liposarcomas (26%) predominated, followed by synovial sarcomas (12%) and malignant fibrous histiocytomas (11%).

**Table I** Presenting symptoms primary.

| *Symptoms* | *Patients* *No.* | % |
|---|---|---|
| Pain | 112 | 43 |
| Mass | 203 | 79 |
| Other | 98 | 38 |
| Menorrhagia or post-menopausal bleeding | 33 | |
| Urinary | 13 | |
| Abdominal distension | 6 | |
| Weight loss | 6 | |
| Respiratory | 8 | |
| Focal weakness | 5 | |

Overall the commonest histological types were leiomyosarcoma (27%), liposarcoma (14%) malignant fibrous histiocytoma (10%) and fibro- and neurofibrosarcomas (15%). Miscellaneous histological types, of which mixed mesodermal and endometrial stromal sarcomas of the uterus were most prevalent, are shown in Table IV.

Histological grade was specified in 186 cases (72%) the majority of cases being high grade (III) – 125 (67%), although 51 (27%) were low grade (I) tumours. Intermediate grade (II) sarcomas were uncommon – 10 (6%).

Clinical size, at presentation of the primary tumour, was recorded in less than half (124) the cases, and accurate measurements were rarely made. Histological reports documented pathological size in 159 cases (62%), but this was not available for inoperable tumours or those removed piecemeal. Sarcomas of unknown size had a higher rate of local recurrence (65% *vs* 50%) and metastasis (73% *vs* 55%) compared with tumours of documented pathological size (Table V). The rate of local recurrence did not seem to vary significantly with pathological size except that only one local relapse was observed in 6 tumours $>400\,cm^2$. Metastases tended to increase in frequency with size up to $225\,cm^2$, with few tumours (22) larger than this available for analysis. Survival was linked to tumour size (Figure 2), those patients with sarcomas $>100\,cms^2$ faring considerably worse ($P<0.01$).

**Table II** Histological type according to site.

| *Histology* | *Head & neck* | *Distal upper limb* | *Proximal upper limb* | *Distal lower limb* | *Proximal lower limb* | *Trunk* | *Retro-peritoneum* | *Visceral* | *Intra-thoracic* | *Other* | *Total No.* | *%* |
|---|---|---|---|---|---|---|---|---|---|---|---|---|
| Angiosarcoma | 3 | 0 | 1 | 0 | 2 | 3 | 1 | 0 | 0 | 0 | 10 | 4 |
| Fibrosarcoma | 1 | 0 | 1 | 1 | 6 | 5 | 1 | 0 | 2 | 0 | 17 | 7 |
| Leiomyosarcoma | 2 | 0 | 2 | 0 | 5 | 3 | 12 | 34 | 4 | 7 | 69 | 27 |
| Liposarcoma | 0 | 1 | 2 | 2 | 19 | 5 | 7 | 1 | 0 | 0 | 37 | 14 |
| Neurofibro-sarcoma | 1 | 0 | 2 | 2 | 5 | 4 | 5 | 0 | 1 | 1 | 21 | 8 |
| Rhabdomyo-sarcoma | 1 | 1 | 3 | 0 | 5 | 2 | 0 | 2 | 0 | 0 | 14 | 6 |
| Synovial sarcoma | 0 | 1 | 1 | 2 | 9 | 0 | 0 | 0 | 0 | 0 | 13 | 5 |
| Undifferentiated | 0 | 0 | 2 | 1 | 6 | 0 | 2 | 1 | 0 | 0 | 12 | 4 |
| Malignant fibrous histiocytoma | 2 | 2 | 5 | 1 | 8 | 4 | 2 | 1 | 0 | 2 | 27 | 10 |
| Miscellaneous | 1 | 0 | 1 | 4 | 8 | 7 | 0 | 15 | 1 | 0 | 37 | 15 |
| TOTAL No. | 11 | 5 | 20 | 13 | 73 | 33 | 30 | 54 | 8 | 10 | 257 | |
| % | 4 | 2 | 8 | 5 | 28 | 13 | 12 | 21 | 3 | 4 | 100 | |

**Table III** Detailed analysis of site.

| | | | |
|---|---|---|---|
| 1. *Head and neck* | 11 | 7. *Retroperitoneum* | 30 |
| Head | 8 | 8. *Visceral* | 54 |
| Neck | 3 | Uterus | 42 |
| 2. *Distal upper limb* | 5 | Stomach | 3 |
| Forearm | 3 | Bowel/mesentery | 3 |
| Hand | 3 | Ovary | 6 |
| 3. *Proximal upper limb* | 20 | 9. *Intrathoracic* | 8 |
| Upper arm | 10 | Lung & pleura | 4 |
| shoulder | 10 | Mediastinum | 4 |
| 4. *Distal lower limb* | 13 | 10. *Miscellaneous* | 10 |
| Lower leg | 8 | Paratesticular | 2 |
| Foot | 5 | Liver | 3 |
| 5. *Proximal lower limb* | 73 | Prostate | 1 |
| Thigh | 56 | Cervical remnant | 1 |
| Buttock | 10 | Broad ligament | 2 |
| Groin | 7 | Multicentric | 1 |
| 6. *Trunk* | 33 | | |
| Breast | 10 | | |
| Perineum | 2 | | |
| Abdominal wall | 3 | | |
| Chest wall | 11 | | |
| Loin/back | 7 | | |

**Table IV** Miscellaneous histology.

| *Histological type* | *No. patients* |
|---|---|
| Mixed mesodermal | 9[a] |
| Endometrial stromal | 5 |
| Unclassfied | 4 |
| Epithelioid | 3 |
| Alveolar rhabdomyosarcoma (adult) | 3 |
| Osteosarcoma soft tissues | 3 |
| Mesenchymoma | 2 |
| Carcinosarcoma | 2 |
| Fibromatosis (metastasising) | 2 |
| Chondrosarcoma soft tissues | 1 |
| Chordoid | 1 |
| Cystosarcoma phyllodes | 1 |
| Ewing's soft tissues | 1 |

[a]6 uterine, 3 ovarian.

**Table V** Pathological size related to local recurrence and metastasis.

| [a]*Pathological size* (*cms*$^2$) | *Local recurrence* *No.* | % | *Metastases* *No.* | % | *Total* *No. patients* |
|---|---|---|---|---|---|
| 0–24 | 19 | 54 | 12 | 34 | 35 |
| 25–99 | 24 | 40 | 34 | 57 | 60 |
| 100–224 | 24 | 57 | 29 | 69 | 42 |
| 225–399 | 11 | 69 | 9 | 56 | 16 |
| 400+ | 1 | 17 | 3 | 50 | 6 |
| TOTAL | 79 | 50 | 87 | 55 | 159 |
| Unknown | 64 | 65 | 72 | 73 | 98 |

[a]Largest diameter multiplied by largest perpendicular diameter.

**Figure 2** Survival according to pathological size.

*Surgical treatment*

Surgical management of the primary tumour prior to referral to the Christie is described in Table VI. Ninety-eight patients had experienced one (72 patients) or more (range 2–11) local recurrences in the intervening period. Amputation was carried out for 34/111 (31%) limb sarcomas.

**Table VI** Surgical treatment of the primary tumour.

| | *Patients* No. | % |
|---|---|---|
| *Resectability* | | |
| Not resectable | 28 | 11 |
| Partially resectable | 43 | 17 |
| Totally resectable | 186 | 72 |
| *Surgical procedure* | | |
| Local resection | 195 | 76 |
| Amputation | 34 | 13 |
| Biopsy only | 26 | 10 |
| [a]Nothing | 2 | 1 |

[a]Histological diagnosis obtained from lymph node metastases.

**Table VII** Clinical trials.

| *Trial* | *Patients entered* No. | % |
|---|---|---|
| *Christie* | | |
| CYVADIC[a] | 22+6[b] | 9 |
| VAC | 19 | 7 |
| IFOS/MTX→VADIC | 11(+2)[c] | 4 |
| Other chemotherapy | 13 | 5 |
| TOTAL | 71 | 28 |
| EORTC | | |
| 62761 | 45 | 17 |
| 62801 | 13 (+3)[c] | 5 |
| 62802 | 13 | 5 |
| 62821 | 10 (+4)[c] | 4 |
| 62771 | 48 | 19 |
| TOTAL | 129 | 50 |
| *No chemotherapy* | 57 | 22 |

[a]These chemotherapeutic reigmes are given in detail in **Tables VIII & VII.**

[b]These patients received CYVADIC scheduled as in the adjvant trial 62771.

[c]Patients relapsing in control arm of adjvant trial 62771 who received chemotherapy.

*Chemotherapy*

Suitable patients were entered into clinical trials evaluating chemotherapy in advanced disease, or as an adjuvant treatment following surgery with or without radiotherapy. Table VII gives the distribution of patients within these trials and Tables VIII and XII describe the chemotherapy protocols for advanced disease and adjuvant therapy respectively.

*Advanced disease*

*Christie studies* Until 1976 patients with advanced disease were treated with the CYVADIC regime (Table VIII). Twenty-five patients received this combination, but pathological material could not be reviewed for three, and there was one early death from malignant disease occuring at 2 weeks. There were 3CR (14%) and 7PR (33%), giving overall response rate of 47%. Durations of remission were: CR 9, 11, 16 months; PR - median 9 months, range 4–27.

In 1982 a small pilot study of alternating chemotherapy (Ifos/MTX VADIC Table VIII) was initiated. Ten consecutive patients were entered into this study, instead of EORTC protocols, and a further 3 patients who lacked clinically measurable disease (but could be evaluated by computed tomography) have been added. There was one early death (3 days) leaving 12 evaluable patients. There have been 2CR, continuing at 16+ and 17+ months from the start of chemotherapy, and 4 PR lasting 9, 3+, 4+ and 14+ months.

*EORTC studies*

Between 1st October 1976 and 1st July 1979 patients with advanced local or metastatic disease were entered into EORTC protocol 62761 comparing two schedulings of the CYVADIC regime (Table VIII). The results of this study have been published elsewhere (Pinedo *et al.*, 1984), and are compared in Table IX with the results from this centre.

Between 1st March 1980 and 25th June 1983 two randomised phase II studies comparing the new anthracyclines Carminomycin (Bramwell *et al.*, 1983) and 4'Epiadriamycin (Mouridsen *et al.*, 1984) with Adriamycin (Table VIII) were completed. Table X compares the results of the multi-centre studies with our own data.

The current first line study initiated 1st October 1982, comparing Cyclophosphamide with Ifosfamide (Table VIII-protocol 62821) includes patients who have received prior chemotherapy, in addition to previously untreated cases. This on-going trial has accrued 134 patients, 77 of whom are previously untreated (March 1984).

A total of 16 patients have been treated with single agent Ifosfamide, as described in Table VIII,

**Table VIII** Chemotherapy regimes for advanced disease

*EORTC* *Protocol 62761*

| *S1—Full CYVADIC* | | | vs | *S2—Split CYVADIC* | | |
|---|---|---|---|---|---|---|
| Cyclophosphamide | 500 mg m$^{-2}$ i.v. | day 1 | | Adriamycin | 50 mg m$^{-2}$ i.v. | day 1 |
| Vincristine | 1.4 mg m$^{-2}$ i.v. | day 1 | | DTIC | 250 mg m$^{-}$2 i.v. | days 1–5 |
| Adriamycin | 50 mg m$^{-2}$ i.v. | day 1 | | Cyclophosphamide | 1200 mg m$^{-2}$ i.v. | day 29 |
| DTIC | 250 mg m$^{-2}$ i.v. | days 1-5 | | Vincristine | 1.4 mg m$^{-2}$ i.v. | day 29 |
| Repeat every 4 weeks | | | | Repeat every 8 weeks | | |

*Protocol 62801*

| | | vs | | |
|---|---|---|---|---|
| Adriamycin | 75 mg m$^{-2}$ i.v. | vs | 4'Epiadriamycin | 75 mg m$^{-2}$ i.v. |
| every 3 weeks | | | every 3 weeks | |

*Protocol 62802*

| | | vs | | |
|---|---|---|---|---|
| Adriamycin | 75 mg m$^{-2}$ i.v. | vs | Carminomycin | 20 mg m$^{-2}$ i.v. |
| every 3 weeks | | | every 3 weeks | |

*Protocol 62821*

| | | vs | | |
|---|---|---|---|---|
| Cyclophosphamide | 2 g m$^{-2}$ i.v. | vs | Ifosfamide | 5 g m$^{-2}$ i.v. |
| as 24 h infusion every 3 weeks | | | as 24 h infusion every 3 weeks | |

*CHRISTIE—CYVADIC*

As S1 regime protocol 62761

*IFOS/MTX→VADIC*—Repeated 1-2 times (omitting VCR)

| | | |
|---|---|---|
| Ifosfamide 24 h infusion | 5 g m$^{-2}$ | days 1, 22, 43 |
| Methotrexate | 250 mg m$^{-2}$ i.v. | days 8, 29, 50 |
| Folinic acid | 15 mg oral 6 hrly × 6 starting at 24 h | |
| Vincristine | 1.4 mg m$^{-2}$ i.v. | days 64, 71, 78, 85, 92, 99 |
| Adriamycin | 50 mg m$^{-2}$ i.v. | days 64, 78, 92 |
| DTIC | 400 mg m$^{-2}$ i.v. | days 64, 65, 78, 79, 92, 93 |

**Table IX** Results EORTC protocol 62761.

| | *S1— FULL CYVADIC* | | | | *S2— SPLIT CYVADIC* | | | |
|---|---|---|---|---|---|---|---|---|
| | *Christie* | | *EORTC* | | *Christie* | | *EORTC* | |
| | *No.* | *%* | *No.* | *%* | *No.* | *%* | *No.* | *%* |
| Entered | 30 | | 125 | | 15[a] | | 121[a] | |
| Evaluable | 27 | | 84 | | 14 | | 78 | |
| CR | 1 | 4 | 14 | 17 | 2 | 14 | 4 | 5 |
| PR | 7 | 26 | 18 | 21 | 1 | 7 | 7 | 9 |
| NC | 8 | 29 | 24 | 29 | 5 | 36 | 35 | 45 |
| PD | 11 | 41 | 28 | 33 | 6 | 43 | 32 | 41 |

[a]This was a randomised trial, but accrual to the S2 arm halted when it was found to be less active. The EORTC figures show only the randomised part of the trial. The Christie figures include all patients, but the results are no different if only randomised patients are considered.

**Table X** Results EORTC protocols 62801, 62802.

| | *Adriamycin* | | | | *4'Epiadriamycin* | | | |
|---|---|---|---|---|---|---|---|---|
| | *Christie* | | *EORTC* | | *Christie* | | *EORTC* | |
| *62801* | *No.* | *%* | *No.* | *%* | *No.* | *%* | *No.* | *%* |
| Entered | 8 | | 106 | | 8 | | 104 | |
| Evaluable | 8 | | 84 | | 7 | | 79 | |
| CR | 0 | 0 | 6 | 7 | 0 | 0 | 2 | 2 |
| PR | 2 | 25 | 15 | 18 | 0 | 0 | 10 | 13 |
| NC | 5 | 62.5 | 39 | 46 | 1 | 14 | 33 | 42 |
| PD | 1 | 12.5 | 24 | 29 | 6 | 86 | 34 | 43 |
| | *Adriamycin* | | | | *Carminomycin* | | | |
| | *Christie* | | *EORTC* | | *Christie* | | *EORTC* | |
| *62802* | *No.* | *%* | *No.* | *%* | *No.* | *%* | *No.* | *%* |
| Entered | 7 | | 42 | | 6 | | 41 | |
| Evaluable | 7 | | 38 | | 6 | | 33 | |
| CR | 0 | 0 | 1 | 3 | 0 | 0 | 0 | 0 |
| PR | 2 | 28.5 | 10 | 26 | 1 | 17 | 1 | 3 |
| NC | 3 | 43 | 18 | 47 | 2 | 33 | 15 | 45 |
| PD | 2 | 28.5 | 9 | 24 | 3 | 50 | 17 | 52 |

of whom 7 were included in EORTC trial 62821. Nine had received prior chemotherapy. There have been 3CR and 5PR, an overall response rate of 50%. Three of the partial remitters have relapsed after 3, 10 and 12 months and one complete remitter died of renal toxicity. Four patients remain in remission although 3 are still receiving chemotherapy.

Table XI summarises the response to first line chemotherapy according to histology. The overall response to first line chemotherapy regimes, single agent and combination, was 34%. No responses were noted in angiosarcomas or synovial sarcomas, although only 4 patients in each group received chemotherapy. Only one partial regression was observed among 8 patients with fibrosarcomas. Undifferentiated (5/8) and liposarcomas (7/14) seemed most responsive, with 3 CR occurring in the latter group. However, the numbers were too small for these differences to be statistically significant.

### Adjuvant trials

### *Christie hospital*

Until 1978, patients with high grade sarcomas, in whom there had been macroscopic removal of the primary tumour, but who were felt to be at high risk for relapse, received 10 cycles of adjuvant chemotherapy with VAC (Table XII). Five year follow up is available on 17 patients, 11 of whom have relapsed, 3 locally, 6 with metastases and 2 with local and metastatic disease. Two patients who experienced local recurrence have been salvaged by amputation and one patient has had lung metastases resected twice and is currently disease free. There have been 8 sarcoma related deaths.

### *EORTC trial*

Protocol 62771 (Pinedo *et al.,* 1979) compares a control group receiving no further treatment with a chemotherapy group receiving CYVADIC (Table XII), after definitive treatment of the primary tumour by surgery and radiotherapy. Since 1st November 1977 a total of 307 patients have been entered into this study, 49 (16%) from the Christie Hospital. Of this latter group, in the control arm there have been 13 relapses in 25 evaluable patients (52%), 3 being local, 9 metastatic and 1 at both sites. Six patients have died of sarcoma and one of pancreatitis (intercurrent death). In the chemotherapy arm there have been 5 relapses in 23 evaluable patients (22%), 2 being local and 3 metastatic; these differences are not significant. Four patients have died of sarcoma and one from infection during a period of chemotherapy-related myelosuppression. Three patients refused chemotherapy after 0,2 and 4 courses and 2 stopped treatment, one because of myelosuppression and the other following a myocardial infarction. There have been 2 relapses among these 5 patients. One patient developed acute myeloid leukaemia 12 months after completing radiotherapy and chemotherapy for a sarcoma of the upper arm. He remains in remission from both sarcoma and leukaemia 26 months later. Chromosomal analysis of leukaemic marrow was normal.

### *Radiotherapy*

Patients entering the EORTC adjuvant trial, 62771 received radical post-operative radiotherapy if there was microscopic residual disease after surgery, or less than 1 cm margin of healthy tissue around the tumour or when a second operation had been

**Table XI** Response according to histological subtype.

| *Histology* | *CR* | *PR* | *SD* | *PD* | *ED* | *Insuff.* | *TE* | *TOTAL* |
|---|---|---|---|---|---|---|---|---|
| Angiosarcoma | 0 | 0 | 1 | 3 | 0 | 0 | 0 | 4 |
| Fibrosarcoma | 0 | 1 | 1 | 4 | 1 | 1 | 0 | 8 |
| Leiomyosarcoma | 5 | 7 | 18 | 9 | 1 | 2 | 0 | 42 |
| Liposarcoma | 3 | 4 | 2 | 4 | 0 | 0 | 1 | 14 |
| Neurofibro-sarcoma | 1 | 2 | 4 | 5 | 0 | 0 | 0 | 12 |
| Rhabdomyo-sarcoma | 1 | 2 | 2 | 4 | 0 | 0 | 0 | 9 |
| Synovial sarcoma | 0 | 0 | 2 | 1 | 0 | 0 | 1 | 4 |
| Undifferentiated | 1 | 4 | 1 | 1 | 1 | 0 | 0 | 8 |
| Malignant fibrous histiocytoma | 1 | 3 | 2 | 4 | 1 | 0 | 1 | 12 |
| Miscellaneous | 2 | 4 | 4 | 7 | 2 | 0 | 0 | 19 |
| TOTAL | 14 | 27 | 37 | 42 | 6 | 3 | 3 | 132[a] |

[a]Number of patients receiving chemotherapy.

**Table XII** Chemotherapy regimes for adjuvant trials

| | | | | |
|---|---|---|---|---|
| *EORTC—Protocol 62771* | | | | |
| *S1—CYVADIC* | | | *vs* | *S2—No chemotherapy* |
| Cyclophosphamide | $500\,mg\,m^{-2}$ i.v. | day1 | | Observation only |
| Vincristine | $1.4\,mg\,m^{-2}$ i.v. | day1 | | |
| Adriamycin | $50\,mg\,m^{-2}$ i.v. | day1 | | |
| DTIC | $400\,mg\,m^{-2}$ i.v. | days 1–3 | | |
| repeat every 4 weeks × 8 | | | | |
| *CHRISTIE—VAC* | | | | |
| Vincristine | $1.4\,mg\,m^{-2}$ i.v. | day 1 | | |
| Adriamycin | $50\,mg\,m^{-2}$ i.v. | day 1 | | |
| Cyclophosphamide | $500\,mg\,m^{-2}$ i.v. | day 1 | | |
| repeat every 3 weeks × 10 | | | | |

performed because the first was considered inadequate. A few patients who were treated before the trial, or were not eligible (eg. age, refusal, long time interval from surgery) also received radical radiotherapy. A total of 51 patients were treated in this way and half have not relapsed. There were 4 local recurrences and 7 patients developed metastases, while both local and distant relapse occurred in 14 patients. An inoperable primary tumour was irradiated in 10 patients, but 8 have progressed locally and 8 are dead. One patient died of metastases, but was locally controlled, one is alive with progressive disease and the remaining patient was treated recently. Palliative irradiation was administered to local recurrences in 43 patients and to mestastases in 41 patients.

*Overall current status*

At the time of analysis 52 patients had never relapsed, of whom 50 were alive and 2 died free of recurrent sarcoma. Ninety-eight (38%) of the 257 patients were alive and 72 (28%) had no evidence of disease. A few patients had stable or partly regressed tumours and advancing disease was evident in 15. A total of 159 patients had died, 147 of these from malignant disease. Three patients died from toxicity – one from infection secondary to myelosuppression, one from renal failure while the remaining patient developed a cardiomyopathy after Adriamycin, followed later by Mitoxantrone. Nine patients died from other causes which included pancreatitis, heart failure, pulmonary embolus, intercurrent infection and convulsions, the exact cause of death being uncertain in two cases. With one exception all had active disease at the time of their demise.

Survival was not influenced by sex, but patients less than 40 years of age did significantly better ($P<0.001$) than older patients. Figure 3 illustrates differences in survival according to site. Patients with tumours of the limbs and trunk fared significantly better than those with visceral tumours. Sarcomas sited in the head and neck, thorax and retroperitoneum were associated with a poor prognosis (overall $P<0.001$). Considering the largest histological groups, survival figures (Figure 4) were better for liposarcomas and malignant fibrous histiocytomas than for leiomyosarcomas and combined neurofibro- and fibrosarcomas, but these differences were not statistically significant ($P=0.22$). Histological grade proved to be the most important prognostic factor, patients with grade I tumours faring significantly better ($P<0.001$) than those with grade II or III sarcomas (Figure 5). Local recurrence (Figure 6) also had an unfavourable association with survival ($P<0.03$).

Considering only low grade tumours (51 cases), males seemed to fare slightly better ($P=0.6$) and the age differences were still significant. Although low grade sarcomas of the trunk and viscera appeared to be associated with a better prognosis than those in the limbs, and survival seemed worse for retroperitoneal and intrathoracic tumours, the numbers in each group were small and these differences were not significant. It is worth comment that all 4 patients with small ($<25\,cm^2$) grade I tumours are alive. There were no significant differences in the other six categories. As in the overall analysis, local recurrence and histological

**Figure 3** Survival according to site.

**Figure 4** Survival according to histological type.

**Figure 5** Survival according to histological grade.

**Figure 6** Survival according to incidence of local recurrence.

subtype did not significantly affect survival. With 4 exceptions low grade tumours were treated by wide resection.

For high grade tumours age, sex, histological subtype and local recurrence did not influence survival. Patients with limb sarcomas had a significantly ($P<0.02$) better prognosis than the remainder (Figure 7) and amputation was associated with a better outcome than local resection (Figure 8). Tumours of increasing size were associated with a worse prognosis ($P<0.002$).

*Patterns of relapse*

At the time of analysis 143 (56%) patients had experienced one or more local recurrences and 159

**Figure 7** Survival in patients with high grade tumours according to site.

**Figure 8** Survival in patients with high grade tumours according to surgical procedure.

(62%) had developed metastatic disease. In 96 cases (37%) both ultimately occurred. Table XIII summarises the incidence of local recurrence and metastasis according to histology. Although neurofibrosarcomas and fibrosarcomas had the highest rate of local recurrence (71%), there were no significant differences between histological types. Rhabdomyosarcomas (79%) synovial sarcomas (79%) undifferentiated (75%) and leiomyosarcomas of the uterus (79%) showed an increased propensity for metastasis, whereas fibrosarcomas (39%) and liposarcomas (43%) metastasised less frequently. All 6 patients with mixed mesodermal sarcomas of the uterus have died of disease, but 4 of 5 patients with endometrial stromal tumours are alive, 3 being disease free.

Table XIV illustrates the pattern of local recurrence and metastasis according to site. Although metastases were less common from sites in the head and neck (45%) and retroperitoneum (43%) local recurrence, (82% and 67% respectively) often not amenable to further surgery, was a particular problem. Visceral sarcomas had a low rate of local recurrence (41%), but a high incidence of metastases (81%). Dissemination was surprisingly frequent in distal lower limb tumours (92%) but this incidence may have been distorted by small numbers.

The sites of first metastasis are summarised in Table XV. A number of patients relapsed at several sites simultaneously. The dominant site of recurrence was pulmonary (57%) followed by intraabdominal disease (17%) usually related to visceral sarcomas. Lymph node metastases occurred in 11% of patients and skin/subcutaneous, bone and liver metastases were also uncommon (8–9%).

**Table XIII** Incidence local recurrence and metastases related to histological type.

| | *Local recurrence* | | *Metastases* | | *TOTAL* | |
|---|---|---|---|---|---|---|
| *Histology* | *No.* | *%* | *No.* | *%* | *No.* | *%* |
| Angiosarcoma | 6 | 60 | 6 | 60 | 10 | 4 |
| Fibrosarcoma | 12 | 71 | 7 | 39 | 17 | 7 |
| Leiomyosarcoma | 35 | 51 | 45 | 66 | 69 | 27 |
| Liposarcoma | 18 | 49 | 16 | 43 | 37 | 14 |
| Neurofibro-sarcoma | 15 | 71 | 14 | 67 | 21 | 8 |
| Rhabdomyosarcoma | 9 | 64 | 11 | 79 | 14 | 5.5 |
| Synovial sarcpma | 8 | 62 | 11 | 79 | 13 | 5 |
| Undifferentiated | 8 | 67 | 9 | 75 | 12 | 4.5 |
| Malignant fibrous histiocytoma | 16 | 59 | 15 | 56 | 27 | 11 |
| Miscellaneous | 16 | 43 | 25 | 66 | 37 | 14 |
| TOTAL | 143 | 56 | 159 | 62 | 257 | 100 |

**Table XIV** Incidence local recurrence and metastases related to site.

| | *Local recurrence* | | *Metastases* | | *Total* | |
|---|---|---|---|---|---|---|
| *Site* | *No.* | *%* | *No.* | *%* | *No.* | *%* |
| Head & neck | 9 | 82 | 5 | 45 | 11 | 4 |
| Distal upper limb | 1 | 20 | 3 | 60 | 5 | 2 |
| Proximal upper limb | 8 | 40 | 11 | 55 | 20 | 8 |
| Distal lower limb | 7 | 54 | 12 | 92 | 13 | 5 |
| Proximal lower limb | 44 | 60 | 43 | 59 | 73 | 28 |
| Trunk | 20 | 61 | 20 | 61 | 33 | 13 |
| Retroperitoneum | 20 | 67 | 13 | 43 | 30 | 12 |
| Visceral | 22 | 41 | 44 | 81 | 54 | 21 |
| Intrathoracic | 5 | 63 | 3 | 38 | 8 | 3 |
| Other | 7 | 70 | 5 | 50 | 10 | 4 |
| TOTAL | 143 | 56 | 159 | 62 | 257 | 100 |

**Table XV** Sites of first metastasis.

| Sites | Patients (159) No.[a] | % |
|---|---|---|
| Lymph nodes | 18 | 11 |
| Skin-subcutaneous | 14 | 9 |
| Bones | 14 | 9 |
| Lungs | 91 | 57 |
| Intra-abdominal | 27 | 17 |
| Liver | 12 | 8 |
| Cerebral | 1 | 0.6 |
| Bone marrow | 1 | 0.6 |
| Other | 7[b] | 4 |

[a]More than one site per patient.
[b]Breast 5 Extradural 2.

### Discussion

Many reviews of large series of sarcomas have appeared in the literature. Such series comprise patients referred to specialist centres for management, and their composition will reflect the major interest of the unit. Thus referral patterns to a department of radiotherapy may differ considerably from those to a department of surgery. Multi-modality management, with collaboration between departments dealing with surgery, radiotherapy and chemotherapy, is increasingly common and some recent series from the United States reflect this policy (Lindberg *et al.*, 1981, Weisenberger *et al.*, 1981 and Rosenberg *et al.*, 1981*b*). The Christie Hospital is a specialist cancer centre dealing predominantly with radiotherapy and chemotherapy. Although specialist surgery is available, this has not generally included the management of sarcomas. Thus the composition of this series will reflect a pattern of referral of patients who might be suitable for chemotherapy or radiotherapy, and consequently will differ considerably from earlier British series (Cade 1951; Windeyer *et al.*, 1966; Coe *et al.*, 1981). Although between 1974 and 1976 comparatively few patients (7–25%) were referred for consideration of adjuvant therapy, this proportion has steadily risen, reaching a peak of 57% in 1982, although the 1983 figures were lower (Figure 1).

The American Joint Committee for Cancer Staging and End-Results (AJC), over an 8 year period, collected a series of 1215 cases of soft sarcoma from 13 institutions within the United States. An anlysis of these cases has been published (Russell *et al.*, 1977) and the present series will be compared with this, with occasional reference to relevant British series.

A male to female ratio of 1.5:1 is unusual, most large series reporting an equal distribution, and there is no clear reason for this bias. The median age, 54 years, is higher than that in the AJC series – 43 years – but agrees with a report of 191 cases collected by the National Soft Tissue Sarcoma Registry (Mettlin *et al.*, 1982) in which the median age was 58.5 years.

Most patients (79%) presented with a mass. In many cases it was painless, but vague aching discomfort often occurred. Severe pain was uncommon, but a limb sarcomas was often associated with neurological deficit due to pressure on major nerves. The high incidence of vaginal bleeding (13%), urinary symptoms and abdominal distension reflects inclusion of a significant proportion (16%) of uterine sarcomas. Although common with metastatic disease, respiratory symptoms from primary sarcomas were rare, but in one patient haemoptysis due to lung metastases led to the discovery of an asymptomatic primary uterine sarcoma. Delay in diagnosis is illustrated by the wide range (0–238 months) and long mean interval (12.4 months) between first symptoms and definitive treatment of the primary tumour, as patients (and sometimes their doctors) often ignored painless lumps until other symptoms supervened.

In comparison with the AJC series and that of Cade (1951) there were fewer extremity tumours, 44% *vs.* 52–53%. Higher proportions of limb sarcomas, 66–71%, were present in two British radiotherapy series (Windeyer *et al.*, 1966, Coe *et al.*, 1981). Visceral sarcomas which comprised the second largest group in our series are not separately classified in the AJC report, and it is not clear whether they have been included with sarcomas of the trunk or completely excluded. Tumours of the thigh and buttock, together with retroperitoneal sarcomas predominate in all large series of soft tissue sarcomas, and this is no exception. In agreement with other series, liposarcomas predominate in the thigh and buttock (26%) and we concur with other findings in the AJC report eg. leiomyosarcoma is the commonest histological type in the retroperitoneum (40%) and synovial sarcoma is predominantly a tumour of the lower extremity – 11 of 13 cases. The four dominant histological types in most early surgical and pathological series (Shiu *et al.*, 1975, Gerner & Moore, 1975 and Rosenberg & Glatstein, 1983) including the AJC study are fibrosarcoma, liposarcoma and rhabdomyosarcoma. Malignant fibrous histiocytoma is becoming increasingly recognised as a separate entity (Weiss & Enzinger 1978; Rantakokko Ekfors, 1979; Kearney *et al.*, 1980), and features in the AJC series. It is likely that many cases previously classified as fibrosarcoma or rhabdomyosarcoma now fall into this group. In 1978 the South West

Oncology Group published the results of a pathological review of 130 cases entered into a chemotherapy trial for advanced disease (Baker & Benjamin 1978). Malignant fibrous histiocytoma (22%), leiomyosarcoma (20%) and fibrosarcoma (10%) were the commonest histological subtypes. The EORTC have found a similar distribution in their CYVADIC study (Pinedo *et al.*, 1984). In view of the high proportion of patients with advanced disease at presentation to the Christie (64%), and the high incidence of visceral sarcomas, it is not surprising that leiomyosarcoma (26%), liposarcoma (14%) and malignant fibrous histiocytoma (11%) are the dominant histological subtypes in this series. Malignant fibrous histiocytoma is probably under-represented as cases in this study have been reviewed over a 10 year time period. Fibrosarcoma, synovial sarcoma and rhabdomyosarcoma were the commonest histological diagnoses among sarcomas of the extremities seen at the Christie in the 20 years preceding this study (unpublished data). In any series biased towards advanced disease, there is likely to be a greater proportion of high grade tumours (67% in this series). Intermediate grade sarcomas were uncommon (6%) and their division from high grade tumours seems to have little clinical significance.

Some studies (Suit *et al.*, 1975; Weiss & Enzinger, 1978, Rantakokko & Ekfors, 1979) have suggested that size is related to prognosis, although others disagree (Markhede *et al.*, 1982). In their proposal for a new staging system, Russell *et al.* (1977) have subdivided stages I to III into A (tumour < 5 cms) and B (> 5 cms), but analysis of their collected cases does not demonstrate a clear survival advantage for smaller tumours. Tumour size was not recorded in approximately one third of cases. A similar problem arose in this series as clinical size was particularly poorly recorded and, for various reasons, pathological size was missing in more than one third of patients. Thus any relationship between size and recurrence/survival must be interpreted with caution. The high incidence of local recurrence and metastases in tumours of unknown size is not surprising as this group includes a large proportion of tumours which could not be resected or only removed piecemeal, which will inevitably recur. The frequency of local recurrence was unrelated to size, possibly because local resection was the commonest surgical procedure, which in many cases took the form of "shelling out", or removal with minimal margins. Often these small tumours were thought to be benign, and even when the true histology was apparent, surgeons were reluctant to reoperate. Unfortunately excisional biopsy may cause quite wide seeding in the operative site, and later radical surgery may still fail. Pre-operative diagnosis of larger sarcomas meant that appropriate initial surgery was more likely, and may account for the suprisingly low incidence of local relapse in tumours > 400 $cm^2$. There were 111 limb sarcomas, of whom 34 (31%) had been treated by amputation at the time of referral to the Christie. In many of these, ablative surgery had been performed after one or more local recurrences. Reviewing the local resections it was clear from the operation notes that compartmental resections was rarely performed. Simon & Enneking (1976) achieved a 98% local control rate in 46 patients with limb sarcomas treated by radical local resection or amputation, and judged to have had an adequate operation. However, amputation was necessary in 29 patients and all eight patients who had inadequate operations experienced local recurrence.

Approximately half the patients in this series were entered into collaborative European trials of chemotherapy, and a further quarter received chemotherapy in local pilot studies. In trials accruing small numbers of patients, there may be considerable variation in response rates due to heterogeneity in patient populations with respect to factors such as age, performance status, histological type and grade and tumour burden. This is illustrated by response rates to the same CYVADIC combination (Table VIII). The overall response rate among all patients entered into EORTC protocol 62761 was 38%, but for Christie patients entering the trial (32% of the total evaluable) it was lower at 30%. In contrast, the response rate in the Christie pilot study was 47%. If all Christie patients are combined the response rate is 37%. Although overall response to the S2 split regime was disappointing, 2 patients with the most durable complete remissions (39, 54 months) received this form of chemotherapy. In the anthracycline studies, the response rate to Adriamcyin among Christie and EORTC trial patients was remarkably consistent between 25–29%. In protocol 62801, there was no significant difference in response rate between 4'Epiadriamycin and Adriamycin, although the former was slightly less toxic (Mouridsen *et al.*, 1984), but we did not obtain any responses in 7 evaluable patients. In contrast we observed the only remission on Carminomycin in protocol 62802 (Table X).

Our response rate (50%) to a new alkylating agent, Ifosfamide, is very encouraging. Even if one complete remitter with Ewing's sarcoma of soft tissues, a highly responsive tumour, is removed the results are similar to these reported from the Royal Marsden (Stuart-Harris *et al.*, 1983). Although the numbers are small, they are supported by data from the 13 patients receiving Ifosfamide in combination with Methotrexate, 6 of whom have responded.

In most chemotherapy trials, no significant differences in response according to histological subtype have emerged because of relatively low response rates and small numbers in each group (Pinedo *et al.*, 1984; Presant *et al.*, 1981; Schoenfeld *et al.*, 1982). Reporting results in 125 patients, Yap *et al.* (1980) found neurofibrosarcoma, rhabdomyosarcoma, leiomyosarcoma, fibrosarcoma and angiosarcoma to be most responsive, which conflicts with our findings. It is likely that in both studies these differences have occurred by chance.

Considering adjuvant chemotherapy, data from the small pilot study of VAC chemotherapy are difficult to interpret. The numbers are small and there is no comparative control group. Patients at high risk for recurrence – eg. tumour spillage at operation, sites where radical resection was difficult, high grade tumours – were given adjuvant therapy, but local relapse has occurred in 29% and metastasis in 47% of cases. The EORTC adjuvant trial 62771 is still accruing patients and it is premature to report results. Among the Christie entries fewer patients have developed metastatic disease in the chemotherapy arm, 3 compared with 9 in the control arm, (although there was one chemotherapy death). This reduced incidence of metastases is in agreement with two randomised studies of adjuvant chemotherapy reported in the United States (Edmonson *et al.*, 1982; Rosenberg *et al.*, 1983), but it would be unwise to draw firm conclusions from a small subgroup of a larger randomised trial which is still in progress. Follow-up is too short to exclude the possibility that postponement of recurrence due to chemotherapy is responsible for the observed difference. Criteria for ensuring that patients were truly disease free at entry into protocol 62771 were much stricter than for the VAC study. Survival is similar in both arms but there have been few events. Difficulties have been encountered in administration of the prescribed chemotherapy, either due to myelosuppression or patients' refusal because of vomiting and alopecia. Although it is possible that our case of acute myeloid leukaemia was induced by chemotherapy, the short time interval, normal karyotype and good response to chemotherapy are atypical features of secondary leukaemia.

Although soft tissue sarcomas have often been considered radioresistant, there are many accounts in the literature of good regressions, and even "cures" of primary tumours, and satisfactory palliation of metastatic disease using radiotherapy (McNeer *et al.*, 1968, Suit & Russell, 1975). In this study the results of irradiating inoperable primary tumours were poor, but some of these were large retroperitoneal tumours placing restrictions on the tolerable dose. The rate of local control following radical post-operative radiotherapy (64%) was disappointing, although similar to another British series (Coe *et al.*, 1981), in comparison with some reports (Rosenberg *et al.*, 1981*b*, Lindberg *et al.*, 1981, Liebel *et al.*, 1981), but this was an unselected group of patients with tumours at all sites. Inadequate prior surgery, often limited to excisional biopsy, may have influenced the results.

The high rate of metastasis (62%) and poor survival (38%) is biased by referral patterns. A few patients were moribund at the time of presentation to the Christie. Remissions in excess of 2 years have been observed in only 4 patients with metastatic disease, one relapse occurring after four and a half years. Unfortunately we have not seen remissions continuing beyond 5 years as reported by Yap *et al.* (1983). The results of current regimes of combination chemotherapy remain disappointing, and new approaches are desparately needed. As soft tissue sarcomas have a propensity for relapse in the lungs, which may be the only site of disease, combined surgery and chemotherapy is often feasible and good results have been reported (Benjamin *et al.*, 1981, Karakousis *et al.*, 1982). To date we have treated 7 patients in this way, 3 of whom are disease free (one requiring a second thoracotomy) although the remaining 4 have relapsed and died. We intent to pursue this line of management in suitable patients.

Rhabdomyosarcomas and synovial sarcomas are usually considered to be aggressive tumours of high metastatic potential (Morton & Eilber, 1982, Enzinger & Weiss 1983). This has been confirmed in this series. For fibrosarcomas, a high rate of local recurrence but reduced incidence of metastases is consistent with other reports (Pritchard *et al.*, 1974, Enzinger & Weiss 1983), but the numbers are small. Although pleomorphic liposarcomas often disseminate, well differentiated and myxoid types are more common and a reduced incidence of metastasis in this series is consistent with other reports (Reszel *et al.*, 1966, Enzinger & Weiss, 1983). Leiomyosarcomas of the uterus, particularly those of high grade, have a dismal prognosis (Spiro & Koss 1965, Hart & Billman 1978), confirmed in this series. Uterine mixed mesodermal sarcomas seemed to do particularly badly whereas endometrial stromal sarcomas fared better, but numbers were small. Salazar and coworkers (1978*b*) showed a higher rate of relapse in mixed mesodermal compared with leiomysarcomas of the uterus, although the former usually presented at a more advanced stage. Problems associated with radical removal account for high rates of local recurrence in the head and neck and retroperitoneum, and many patients probably die of local disease before they can develop metastases.

In this study, the most significant factor influencing survival was histological grade, a finding that has been extensively documented in the literature (Markhede *et al.*, 1982, Rantakokko & Ekfors, 1979; Russell *et al.*, 1977; Shiu *et al.*, 1975, Rosenberg & Glatstein, 1983 and Suit *et al.*, 1975). Russell *et al.* (1977), reporting an analysis of the AJC series of 1215 cases, proposed a staging system principally based on this factor. The other significant prognostic factors in this series were primary site and size. As limb and visceral sarcomas are more amenable to radical resection than tumours of the head, neck and trunk, their better prognosis is not surprising and is well documented (Lindberg *et al.*, 1975; Rosenberg *et al.*, 1981*b*). The relevance of size has been discussed earlier. Local recurrence was correlated with a poorer survival and is in agreement with much of the published literature. The finding that histological subtype is unimportant, particularly when the influence of grade is removed, is consistent with the published literature, and reflects uncertainties in determining histogenesis in many tumours. For high grade tumours amputation was associated with a better outcome than local resection. The number of patients with low grade tumours treated by amputation was too small to assess the relative merits of these procedures. The better overall survival for younger individuals was not evident for high grade tumours. Younger patients may be more likely to receive radical treatment which may influence survival for low grade tumours. A high rate of metastasis in grade III tumours, with ultimate demise of the patient, is unlikely to be related to age.

As anticipated the commonest first site of metastasis was pulmonary, occurring in 58% of cases. Intraabdominal disease usually took the form of peritoneal and omental seeding from visceral tumours, although it was not always possible to determine the exact sites of tumour. At least initially, other sites such as lymph nodes, liver and bone were less common, although we know from post mortem studies that utlimately widespread dissemination may occur (Kavanagh 1980) and even cerebral metastases are more common after chemotherapy (Bryant & Wiltshaw 1980; Espana *et al.*, 1980).

We would like to thank Gillian Wood and Vivien Forsythe for invaluable assistance with data handling, and Eileen Morgan and Barbara Whittle for typing the manuscript.

### References

BAKER, L.H. & BENJAMIN, H.S. (1978). Histological frequency of disseminated soft tissue sarcoma in adults. *Proc. Am. Soc. Clin. Onc.*, **19**, 324.

BAKER, L., BENJAMIN, R., FINE, G. SAIKI, J. & RIVKIN, S. (1979). Combination chemotherapy in the management of disseminated soft tissue sarcomas – a Southwest Oncology Group (SWOG) study. *Proc. Am. Soc. Clin. Oncol.*, **20**, 378.

BENJAMIN, R., YAP, B., FRAZIER, O. BODEY, G. (1981). Combination chemotherapy for sarcomas with cyclophosphamide and continuous infusion adriamycin and dacarbazine (CI-CYVADIC) with surgical intensification. *Proc. Am. Soc. Clin. Oncol.*, **22**, 526.

BORDEN, E.C., AMATO, D., ENTERLINE, H.T., LERNER, H. & CARBONE, P.P. (1983). Randomised comparison of adriamycin regimens for treatment of metastatic soft tissue sarcomas. *Proc. Am. Soc. Clin. Oncol.*, **24**, 231.

BRAMWELL, V.H.C., MOURIDSEN, H.T., MULDER, J.H. & 6 others (1983). Carminomycin vs adriamycin in advanced soft tissue sarcomas: an EORTC randomised phase II study. *Eur. J. Cancer Clin. Oncol.*, **29**, 1097.

BRAUND, R.R. & PIGOTT, J.D. (1962). Soft tissue sarcomas of the head and neck. *Am. J. Surg.*, **104**, 732.

BRYANT, B.M. & WILTSHAW, E. (1980). Central nervous system involvement in sarcomas. *Eur. J. Cancer*, **16**, 1503.

CADE, S. (1951). Soft tissue tumours: their natural history and treatment. *Proc. R. Soc. Med.*, **44**, 19.

CODY, H.S., TURNBULL, A.D., FORTNER, J.G. & HADJU, S.I. (1981). The continuing challenge of retroperitoneal sarcomas. *Cancer*, **47**, 2147.

COE, M.A., MADDEN, F.J. & MOULD, R.F. (1981). The role of radiotherapy in the treatment of soft tissue sarcoma: a retrospective study, 1958–73. *Clin. Rad.*, **32**, 47.

DAS GUPTA, J.K., PATEL, M.K., CHAUDHURI, P.K. & BRIELE, H.A. (1982). The role of chemotherapy as an adjuvant to surgery in the initial treatment of primary soft tissue sarcomas in adults. *J. Surg. Oncol.*, **19**, 139.

EDMONSON, J.H., FLEMING, T.R., IVINS, J.C. & 5 others (1982). Randomised study of systemic chemotherapy following complete excision of non-osseous sarcomas: interim report. *Proc. Am. Soc. Clin. Oncol.*, **1**, 182.

ENZINGER, F.M. & WEISS, S.W. (1983). Soft tissue tumours. C.V. Mosby, 1983.

ESPANA, P., CHANG, P. & WIERNIK, P.H. (1980). Increased incidence of brain metastases in sarcoma patients. *Cancer*, **45**, 377.

GERNER, R.E. & MOORE, G.E. (1975). Synovial sarcoma. *Ann. Surg.*, **181**, 22.

GERSON, R., SHIU, M.H. & HADJU, S.I. (1982). Sarcoma of the buttock: a trend toward limb-saving resection. *J. Surg. Oncol.*, **19**, 238.

GOTTLIEB, J.A., BAKER, L.H., O'BRYAN, R.M. & 15 others (1975). Adriamycin (NSC-123127) used alone and in combination for soft tissue and bone sarcomas. *Cancer Chemother. Rep.*, **6**, 271.

HART, W.R. & BILLMAN, J.K. (1978). A reassessment of uterine neoplasms originally diagnosed as leiomyosarcomas. *Cancer*, **41**, 1902.

KARAKOUSIS, C.P., RAO, U. &. PARK, H.C. (1982). Combination chemotherapy (CYVADIC) in metastatic soft tissue sarcomas. *Eur. J. Cancer Clin. Oncol.*, **18**, 33.

KAVANAGH, J., YAP, B., LUNA, M. & TASHIMA, C. (1980). Metastatic patterns of adult soft tissue sarcomas (ASTS). *Proc. Am. Soc. Clin. Oncol.*, **21**, 480.

KEARNEY, M.M., SOULE, E.H. & IVINS, J.C. (1980). Malignant fibrous histiocytoma: a retrospective study of 167 cases. *Cancer*, **45**, 167.

LEIBEL, S.A., TRANBAUGH, R.F., WARA, W.M. & 6 others. (1981). Soft tissue sarcomas of the extremities: Survival and patterns of failure with conservative surgery and post-operative irradiation compared to surgery alone. *Int. J. Rad. Oncol. Biol. Phys.*, **7**, 1252.

LINDBERG, R.D., MARTIN, R.G. & ROMSDAHL, M.M. (1975). Surgery and post-operative radiotherapy in the treatment of soft tissue sarcomas in adults. *Am. J. Roent. & Rad. Therapy & Nucl. Med.*, **123**, 123.

LINDBERG, R.D., MARTIN, R.G., ROMSDAHL, M.M. & BARKLEY, H.T. (1981). Conservative surgery and post-operative radiotherapy in 300 adults with soft tissue sarcomas. *Cancer*, **47**, 2391.

MARKHEDE, G., ANGERVALL, L. & STENER, B. (1982). A multivariate analysis of the prognosis after surgical treatment of malignant soft tissue tumours. *Cancer*, **49**, 1721.

McNEER, G.P., CANTIN, J., CHI, F. & NICKSON, J.J. (1968). Effectiveness of radiation therapy in the management of sarcoma of the soft somatic tissues. *Cancer*, **22**, 391.

METTLIN, C., PRIORE, R., RAO, U., GAMBLE, D., LANE, W. & MURPHY, G.P. (1982). Results of the National Soft Tissue Sarcoma Registry. *J. Surg. Oncol.*, **19**, 224.

MORTON, D.L. & EILBER, F.E. (1982). Soft tissue sarcomas Section XXXI. In *Cancer Medicine* (Ed. Holland, Frei) Lea & Fabiger. p. 2141.

MOURIDSEN, H.T., SOMMERS, R., SANTORO, A. & 6 others. (1984). Adriamycin versus 4'Epiadriamycin in advanced soft tissue sarcomas. An EORTC Randomised Phase II study. In *Advances in Anthracycline Therapy: Epirubicin'*. (Ed. Bonadonna). (In press).

PETO, R., PIKE, M.C., ARMITEGE, P. & 7 others. (1975). Design and analysis of randomised clinical trials requiring prolonged observation of each patient. *Br. J. Cancer*, **35**, 1.

PINEDO, H.M., BRAMWELL, V.H.C., MOURIDSEN, H.T. & 10 others. (1984). CYVADIC in advanced soft tissue sarcoma: A randomised study with two schedules. *Cancer* (In press).

PINEDO, H.M., VENDRIK, C.P.J., BRAMWELL, V.H.C. & 6 others. (1979). Evaluation of adjuvant therapy in soft tissue sarcoma: A collaborative multidisciplinary approach (EORTC protocol 62771). *Eur. J. Cancer*, **15**, 811.

PRESANT, C.A., LOWENBRAUN, S., BARTOLUCCI, A.A., SMALLEY, R.V. & THE SOUTH EASTERN CANCER STUDY GROUP. (1981). Metastatic sarcomas: Chemotherapy with Adriamycin, Cyclophosphamide and Methotrexate alternating with Actinomycin D., DTIC and Vincristine. *Cancer*, **47**, 457.

PRITCHARD, D.J., SOULE, E.H., TAYLOR, W.F. & IVINS, J.C. (1974). Fibrosarcoma – a clinicopathologic and statistical study of 199 tumours of the soft tissues of the extremities and trunk. *Cancer*, **33**, 888.

RANTAKOKKO, V. & EKFORS, O. (1979). Sarcomas of the soft tissues in the extremities and limb girdles. *Acta Chir. Scand.*, **145**, 385.

RESZEL, P.A., SOULE, E.H., COVENTRY, M.B. (1966). Liposarcoma of the extremities and limb girdles: A study of 222 cases. *J. Bone Joint Surg.*, **48-A**, 229.

ROSENBERG, S.A. & GLATSTEIN, E. (1983). The management of local and regional soft tissue sarcomas. Chapter 78. In *Principles of Cancer Treatment*. Ed. (Carter *et al.*) McGraw-Hill. p. 697.

ROSENBERG, S.A., TEPPER, J., GLATSTEIN, E. & 8 others. (1982). The treatment of soft tissue sarcomas of the extremities: Prospective randomised evaluations of (1) limb-sparing surgery plus radiation therapy compared with amputation and (2) the role of adjuvant chemotherapy. *Ann. Surg.*, **196**, 305–315.

ROSENBERG, S.A., TEPPER, J., GLATSTEIN, E. & 9 others. (1983). Prospective randomised evaluation of adjuvant chemotheapy in adults with soft tissue sarcomas of the extremities. *Cancer*, **52**, 424.

ROSENBERG, S.A., TEPPER, J. GLATSTEIN, E. & 4 others. (1981*a*). Local control of soft tissue sarcomas of the extremities: Preliminary analysis of a prospective randomised trial. *Proc. Am. Soc. Clin. Oncol.*, **22**, 529.

ROSENBERG, S.A., TEPPER, J., GLATSTEIN, E. & 4 others. (1981*b*). Adjuvant chemotherapy for patients with soft tissue sarcomas. *Surg. Clin. N. Am.*, **61**, 1415.

RUSSELL, W.O., COHEN, J., ENZINGER, F. & 7 others. (1977). A clinical and pathological staging system for soft tissue sarcomas. *Cancer*, **40**, 1562.

SAIKI, J.H., RIVKIN, S.E., BAKER, L.H., SHAHBENDER, S. & FLETCHER, W.S. (1982). Adriamycin and single dose DTIC in soft tissue and bone sarcomas – a Southwest Oncology Group Study. *Proc. Am. Soc. Clin. Oncol.*, **1**, 181.

SALAZAR, O.M., BONFIGLIO, T.A, PATTERN, S.F. & 4 others. (1978*a*). Uterine sarcomas: Natural history, treatment and prognosis. *Cancer*, **42**, 1152.

SALAZAR, O.M., BONFIGLIO, T.A., PATTEN, S.F. & 4 others. (1978*b*). Uterine sarcomas: Analysis of failures with special emphasis on the use of adjuvant radiation therapy. *Cancer*, **42**, 1161.

SCHOENFELD, D.A., ROSENBAUM, C., HORTON, J., WOLTER, J.M., FALKSON, G. & DECONTI, R.C. (1982). A comparison of adriamycin versus vincristine and adriamycin, and cyclophosphamide versus vincristine, actinomycin-D and cyclophosphamide for advanced sarcoma. *Cancer*, **50**, 2757.

SHIU, M.H., CASTRO, E.B., HADJU, S.I. & FORTNER, J.G. (1975). Surgical treatment of 279 soft tissue sarcomas of the lower extremity. *Ann. Surg.*, **182**, 597.

SIMON, M.A. & ENNEKING, W.F. (1976). The management of soft tissue sarcomas of the extremities. *J. Bone Joint Surg.*, **58A**, 317.

SORDILLO, P.P., MAGILL, G.B., SHIU, M.H., LESSER., M. HADJU, S.I. & GOLDBEY, R.B. (1981). Adjuvant chemotherapy of soft tissue sarcomas with ALOMAD (S4). *J. Surg. Oncol.*, **18**, 345.

SPIRO, R.H. & KOSS, L.G. (1965). Myosarcoma of the uterus: A clinico-pathological study. *Cancer*, **18**, 571.

SPITTLE, M.F., NEWTON, K.A. & MACKENZIE, D.H. (1971). Liposarcoma: A review of 60 cases. *Br. J. Cancer*, **24**, 696.

STUART-HARRIS, R.C., HARPER, P.G., PARSONS, C.A. & 4 others. (1983). High dose alkylation therapy using ifosfamide infusion with mesna in the treatment of adult advanced soft tissue sarcoma. *Cancer Chem. Pharmacol.*, **11**, 69.

SUIT, H.D., PROPPE, K.H., MANKIN, H.J. & WOODS, U.C. (1981). Pre-operative radiation therapy for sarcoma of soft tissue. *Cancer*, **47**, 2269.

SUIT, H.D. & RUSSELL, W.O. (1975). Radition therapy of soft tissue sarcomas. *Cancer*, **36**, 759.

SUIT, H.D., RUSSELL, W.O. & MARTIN, R.G. (1975). Sarcoma of soft tissue: Clinical and histopathological parameters and response to treatment. *Cancer*, **35**, 1478.

WEISENBURGER, T.H., EILBER, F.R., GRANT, T.T. & 4 others. (1981). Multidisciplinary 'limb salvage' treatment of soft tissue and skeletal sarcomas. *Int. J. Rad. Oncol. Biol. Phys.*, **7**, 1495.

WEISS, S.W. & ENZINGER, F.M. (1978). Malignant fibrous histiocytoma: An analysis of 200 cases. *Cancer*, **41**, 2250.

WILTSHAW, E., HARMER, C. & MCKINNA, A. (1979). Soft tissue sarcoma: Treatment of advanced diseasse in the Royal Marsden Hospital. In: *International Course on Recent Advances in the Treatment of Ovarian and Testicular Cancer and of Soft Tissue and Bone Sarcomas.* Noordwijkerhout, Netherlands. Dec. 1979.

WINDEYER, B., DISCHE, S. & MANSFIELD, C.M. (1966). The place of radiotherapy in the management of fibrosarcoma of soft tissues. *Clin. Radiol.*, **17**, 32.

YAP, B.S., BAKER, L.H., SINKOVICS, J.G. & 6 others. (1980). Cyclophosphamide, vincristine, adriamycin and DTIC (CYVADIC) combination chemotherapy for the treatment of advanced sarcomas. *Cancer Treat. Rep.*, **64**, 93.

YAP, B.S., BURGESS, M.A., SINKOVICS, J.G., BENJAMIN, R.S. & BODEY, G.P. (1981). A five year experience with cyclophosphamide, vincristine, adriamycin and DTIC (CYVADIC) chemotherapy in 169 adults with advanced soft tissue sarcoma (ASTS). *Proc. Am. Soc. Clin. Oncol.*, **22**, 534.

YAP, B.S., SINKOVICS, J.G., BURGESS, M.A., BENJAMIN, R.S. & BODEY, G.P. (1983). The curability of advanced soft tissue sarcomas in adults with chemotherapy. *Proc. Am. Soc. Clin. Oncol.*, **2**, 239.